# Articulatory effort modulates speech adaptation to auditory perturbations

**DOI:** 10.3389/fnhum.2026.1756554

**Published:** 2026-05-22

**Authors:** Elodie Ronayette, Fabien Cignetti, Pascal Perrier, Maëva Garnier

**Affiliations:** 1 Université Grenoble-Alpes, CNRS, Grenoble-INP, GIPSA-Lab, Grenoble, France; 2 Univ. Grenoble Alpes, Inserm U1216, Grenoble Institut Neurosciences, Grenoble, France

**Keywords:** articulatory effort, auditory-motor adaptation, electromyography, formant shift, lip tube, speech

## Abstract

Speech sensorimotor adaptation is typically partial, varies across individuals, and often saturates under large auditory perturbations. While sensory and phonological factors have been proposed to explain this variability, the role of motor effort and its influence on the compensatory response remain largely unexplored. Our study examined whether the physical effort involved in producing compensatory gestures influences both the overall magnitude of adaptation and the perturbation level at which this adaptation begins to saturate. Native French speakers produced the minimal pair /ne/–/nø/ while receiving gradually upshifted second-formant (F2) feedback (0–50%) in two conditions using deformable lip tubes: a very flexible tube (FLT) and a more rigid tube (RLT), both allowing lip rounding but increasing the effort required to achieve it. Compensation was quantified acoustically (F2 decrease) and physiologically (increase in EMG activity of the orbicularis oris muscle) in 21 participants. F2 compensation increased with perturbation level but saturated earlier and at a lower magnitude when articulation required greater effort (RLT). Lip-muscle activity showed a similar nonlinear pattern, with saturation in EMG and F2 occurring at comparable perturbation levels. Variations in F1 suggested that most participants initially relied on increased lip rounding, but some shifted at higher perturbation levels toward another articulatory strategy very likely to involve tongue backing. These results show that speech adaptation depends not only on error monitoring and correction but is also constrained by the physical effort required to produce compensatory movements, which both limits the magnitude of adaptation and influences the selection of compensatory strategies.

## 1 Introduction

Numerous studies have investigated how individuals adapt their speech production in response to altered auditory feedback, compensating in the direction opposite to the imposed perturbation [see Caudrelier and Rochet-Capellan (2019) for a review]. For instance, speakers tend to lower their pitch when they receive upward-shifted pitch in the auditory feedback of their own voice (Jones and Munhall, 2000), or to produce vowels with higher first formant frequencies (F1) when their auditory feedback undergoes a spectral transformation that lowers F1 (Houde and Jordan, 1998). Such compensation is observed in the majority of participants, as early as 150 ms after the perturbation onset (Burnett et al., 1997; Cai et al., 2011), even when the perturbation occurs only once and unpredictably, although in that case with a longer latency (~400 ms; Purcell and Munhall, 2006b). The speakers are not consciously aware of the perturbation or their compensatory response, and they continue to compensate even when they are instructed to ignore the feedback or to avoid compensation (Munhall et al., 2009; Keough et al., 2013; Mitsuya et al., 2015; Kim and Max, 2021; Parrell and Niziolek, 2021; Ning, 2022). This reflects a regulatory mechanism whereby an intended speech production is compared to the actual output via sensory feedback: when a discrepancy is detected between the intended speech and the produced speech, an immediate motor adjustment is triggered to reach a better correspondence between the two (Houde and Jordan, 1998; Villacorta et al., 2007).

For longer-lasting or repeated perturbations involving formant shifts, the degree of compensation typically increases rapidly over the first few utterances produced under altered feedback, and then stabilizes at a stable compensatory level (MacDonald et al., 2012). This compensatory response is only partial and varies across individuals and vowels, typically reaching up to 30% of the perturbation magnitude for the vowel [ɛ] (Purcell and Munhall, 2006b; Munhall et al., 2009; MacDonald et al., 2010; Mitsuya et al., 2015; Trudeau-Fisette et al., 2017). Under these kinds of perturbations, when the perturbation is removed, speech production does not return immediately to the baseline. Instead, compensation persists for some time, and then progressively fades until the baseline production is restored (Purcell and Munhall, 2006a; Villacorta et al., 2007; Shiller et al., 2009). In parallel, impacts on speech perception are also observed in the participants following motor learning with altered auditory feedback (Ghosh et al., 2010; Lametti et al., 2014). There is also evidence of faster re-adaptation, or savings, when individuals are later re-exposed to the same perturbation (Rao and Ostry, 2025). All these phenomena are fundamental characteristics of sensorimotor learning, and could reflect the updating of internal representations involved in speech motor control. In particular, they may arise from adaptive changes in internal models: inverse models, which map desired sensory outcomes to appropriate motor commands, or forward (direct) models, which predict the sensory consequences of those commands (Wolpert et al., 1998; Tourville and Guenther, 2011; Patri et al., 2018).

The degree of compensation shows substantial variability across individuals and contexts (Caudrelier and Rochet-Capellan, 2019). Among the different factors of influence, previous research has shown that the compensatory response depends on the perturbation magnitude, with a saturation effect—defined as a slowing down, plateauing, or even a reversal of changes in the compensatory parameter—at high levels of auditory perturbation. This saturation effect happens in response to formant shifts (MacDonald et al., 2010; Katseff et al., 2012) as well as pitch shifts (Liu and Larson, 2007; Liu et al., 2010; Scheerer et al., 2013). It has also been shown that the compensatory response depends on the direction of the perturbation, with stronger compensatory responses observed for downward pitch perturbations (Liu et al., 2011) and for upward formant perturbations (Villacorta et al., 2007), compared to perturbations of equal magnitude in the opposite direction.

Phonological explanations for saturation and asymmetry have been suggested. Interestingly, these phenomena occur regardless of whether the auditory perturbation or the compensatory gesture remains within or exceeds the boundaries of the sensory targets associated with the phonological category (Niziolek and Guenther, 2013). Such saturation and asymmetry phenomena are observed not only for auditory perturbations that affect categorical perception (formant-shift, or pitch-shift in tonal languages; Liu et al., 2010) but also for perturbations that do not affect phoneme categorization [pitch-shift in non-tonal languages (Burnett et al., 1997) or manipulations of feedback intensity like in the Lombard or sidetone effects (Garnier et al., 2006)]. It suggests that phonological boundaries alone cannot fully account for these saturation and asymmetry effects, and that non-linguistic factors may also play a role, such as mechanical or physiological constraints on the underlying articulatory movements. Indeed, adaptation to increasing levels of perturbation may be limited at some point by the available range of motion amplitude, shaped by the speaker’s anatomy and motor capacities, as well as the physical effort required to carry out the compensatory movement. Thus, adaptation to upward or downward shifted auditory feedback may involve a similar degree of acoustic compensation, but compensatory gestures elicited may require different articulatory constraints and physiological effort. The partial correction of perturbations might also reflect a compromise between target reaching and biomechanical constraints, meaning that the adaptation effort is perceived as disproportionate compared to the benefit for communication.

In optimal control theory, movement is modeled as the outcome of an optimization process in which the nervous system minimizes a cost function such as the peak of velocity, the jerk (i.e., the rate of change of acceleration over time), the target approach speed, the level of force or torque, or the amount of change in neural motor commands (Nelson, 1983; Hogan, 1984; Uno et al., 1989; Jeannerod, 1990). This is particularly the case in the optimal feedback control (OFC) models (Todorov and Jordan, 2002; Guigon et al., 2007; Haith and Krakauer, 2013). This optimization of control can therefore be interpreted as minimizing a motor effort. Effort relates to underlying energetic expenditure, as suggested by formulations that explicitly link effort to metabolic energy consumption (Shadmehr et al., 2016). Within this optimal framework, the nervous system is thought to optimize the trade-off between task accuracy, precision, stability and effort, producing actions that are efficient given task demands (Liu and Todorov, 2007; Cos et al., 2011). Beyond effort minimization, movements might also be shaped by utility optimization, whereby the nervous system evaluates potential actions based on a combination of expected rewards and effort costs. In this view, motor behavior reflects the selection of actions that maximize a utility function, integrating factors such as energetic cost, task success, and reward rate (Körding et al., 2004; Shadmehr et al., 2016; Gordon et al., 2021). Importantly, motor adaptation to novel environments can be viewed as a process of re-optimization, in which new movement plans are formed to minimize motor costs (e.g., effort) while maximizing rewards (Emken et al., 2007; Izawa et al., 2008; Braun et al., 2009).

These principles of optimal control and effort minimization extend naturally to speech production, where movements rely on the coordinated activity of multiple effectors, making the notion of effort inherently multifaceted. Yet, to our knowledge, only a few speech motor control models explicitly integrate the concept of effort. These models aim to optimize speech production by achieving intended auditory targets (Bailly, 1997; Patri et al., 2015; Rakotomalala et al., 2025) or high intelligibility (Elie et al., 2024), while minimizing measures of effort such as jerk, target-approach speed, or the magnitude of changes in motor commands. In these frameworks, effort is treated as a criterion contributing to the selection of motor commands once the sequence of motor goals—here, the sequence of articulatory and acoustic features associated with the phonemes of a given speech task—has been determined. However, they do not consider the possible role of effort at a higher cognitive level, such as in the selection of the motor goals themselves (e.g., choosing among alternative articulatory or acoustic realizations of a same phoneme, or even among different phonemes or words that achieve a same communicative goal). Yet, just as individuals adapt their arm movements to visual perturbations by selecting the least effortful strategy (Cos et al., 2011), it is expected that individuals also adapt their speech to auditory perturbations by choosing strategies—both in terms of articulatory gestures and the magnitude of compensation—that optimize the trade-off between speech intelligibility and biomechanical cost.

To contribute to this question, our study examines how the physical effort involved in producing compensatory gestures influences both the overall magnitude of adaptation and the perturbation level at which this adaptation begins to saturate. Participants produced the rounded vowel /ø/ under F2 upward-shifted auditory feedback. The effort associated with the compensatory lip-rounding gesture was both quantified from the electromyographic activity of the orbicularis oris muscle, and experimentally manipulated, by having participants produce the vowels using two deformable lip tubes with different levels of rigidity. We predicted that increasing the mechanical resistance of the compensatory gesture would reduce the magnitude of acoustic compensation (H1a) and cause the acoustic compensation to begin saturating at lower perturbation levels (H1b). We further hypothesized that this saturation, and its occurrence at lower or higher levels of auditory perturbation, would reflect physical effort—operationalized here as the magnitude of neuromuscular activation—reaching a functional threshold or maximum (H2).

## 2 Material and method

### 2.1 Participants

The study included 36 healthy native French speakers (including 18 men, aged 26 ± 4 years). Participants reported no history of neurological, sensory, speech, or language disorders. Informed consent was obtained from all participants prior to the experiment. The project was approved by the ethics committee of the University Grenoble Alpes (CERGA-Avis-2024-05).

### 2.2 Experimental procedure

#### 2.2.1 Main task

**Figure 1:**
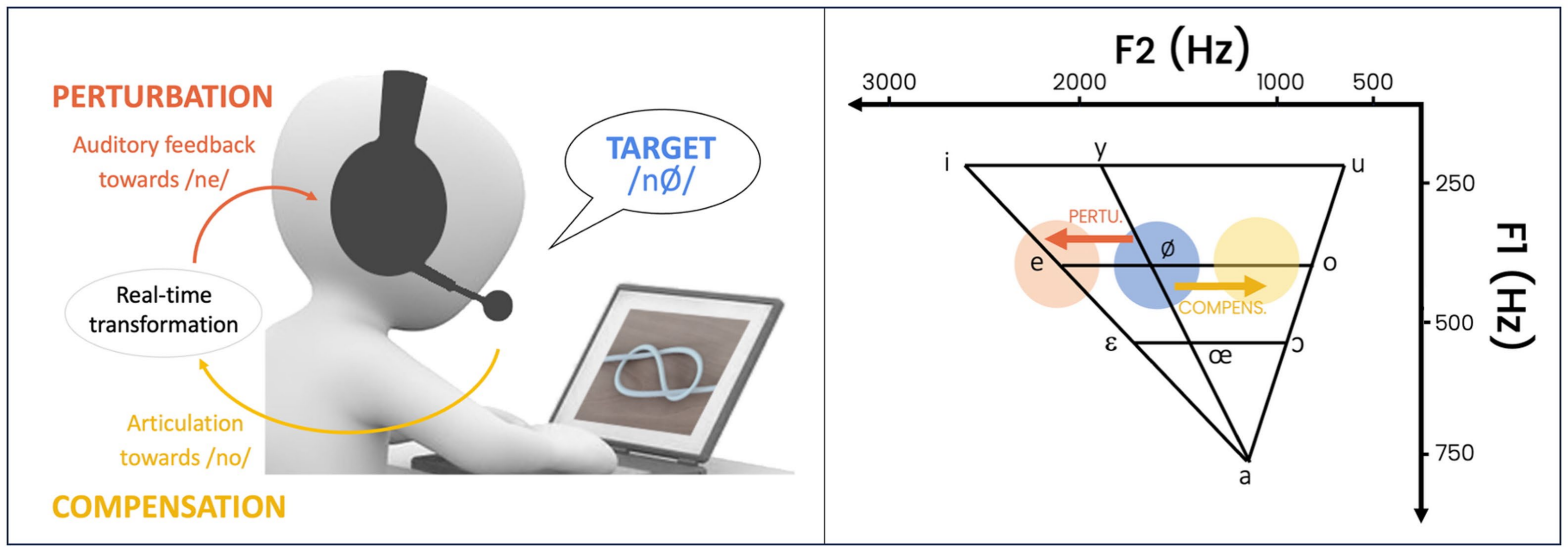
Real-time upward shift of the second formant (F2) in the participants’ auditory feedback of their own voice, during the production of vowel /ø/ (“Target”), shifting their perception toward /e/ (“Perturbation”), and requiring them to modify their articulation toward /o/ (“Compensation”).

Participants performed a picture-naming task involving the production of the target word “nœud” /nø/ (“knot” in French), presented in random alternation with its minimal pair “nez” /ne/ (“nose” in French). This latter was not analyzed in this study but served as a distractor and encouraged participants to maintain the /e/–/ø/ phonological contrast. Participants were instructed to pronounce the appropriate word within 1.5 s after the image was displayed on a screen, in front of them (see Figure 1).

#### 2.2.2 Real-time perturbation of the auditory feedback

Participants’ voices were recorded by a microphone positioned 2 cm from the lips, and they received real-time auditory feedback of their own voice through headphones (see Figure 1). The voice feedback was calibrated at a comfortable listening level of 80 dB, and mixed with a 60 dB pink noise, to mask bone-conducted feedback. The voice feedback was either played back unaltered or with a real-time (delay <20 ms) upward shift of the second formant (F2) applied to the entire produced word, using the software Audapter v2.1.012 (Cai et al., 2008; Tourville et al., 2013). Formants estimation in Audapter used prior values of the first two formants (fn1 and fn2 parameters) that were adjusted according to the target French vowel /ø/ and the participant’s gender (Tubach, 1989).

The upward shift of the second formant (F2) in vowel /ø/ was expected to shift participants’ perception toward the vowel /e/, thereby inducing a lexical confusion between the word “noeud” and its competitor “nez” (see Figure 1). In response to this alteration of auditory feedback, participants were expected to adjust their vowel articulation so as to decrease F2, that is, in the direction of the vowel /o/.

Each experimental condition consisted of a sequence of 16 blocks, each comprising 15 trials (a random alternation of 10 occurrences of /nø/ and 5 of /ne/). Blocks were separated by a 7-s break to allow participants to swallow if necessary. After six baseline blocks with unaltered auditory feedback, participants were exposed to gradually increasing real-time perturbation of the second formant (F2), shifted by +5% at each block, progressively transforming the vowel /ø/ toward /e/. Ten perturbation levels were explored from 5 to 50% (5, 10, 15, 20, 25, 30, 35, 40, 45, and 50%). A gradual increase was chosen to keep the auditory perturbation below participants’ awareness and to elicit implicit adaptation for as long as possible. The purpose of testing such a wide range of perturbation levels—extending beyond those typically used in previous studies (around 35% on average)—was to observe possible saturation effects in speech compensation, as reported in previous studies with increasingly pitch-shifted (Liu and Larson, 2007; Liu et al., 2010; Scheerer et al., 2013) or F1-shifted feedback (MacDonald et al., 2010; Katseff et al., 2012). In that case, we aimed to determine whether saturation might occur earlier, i.e., at lower perturbation level, when the compensatory gesture requires greater physical effort. The succession of 16 such blocks lasted approximately 10 min.

#### 2.2.3 Manipulation of lip-rounding effort

**Figure 2:**
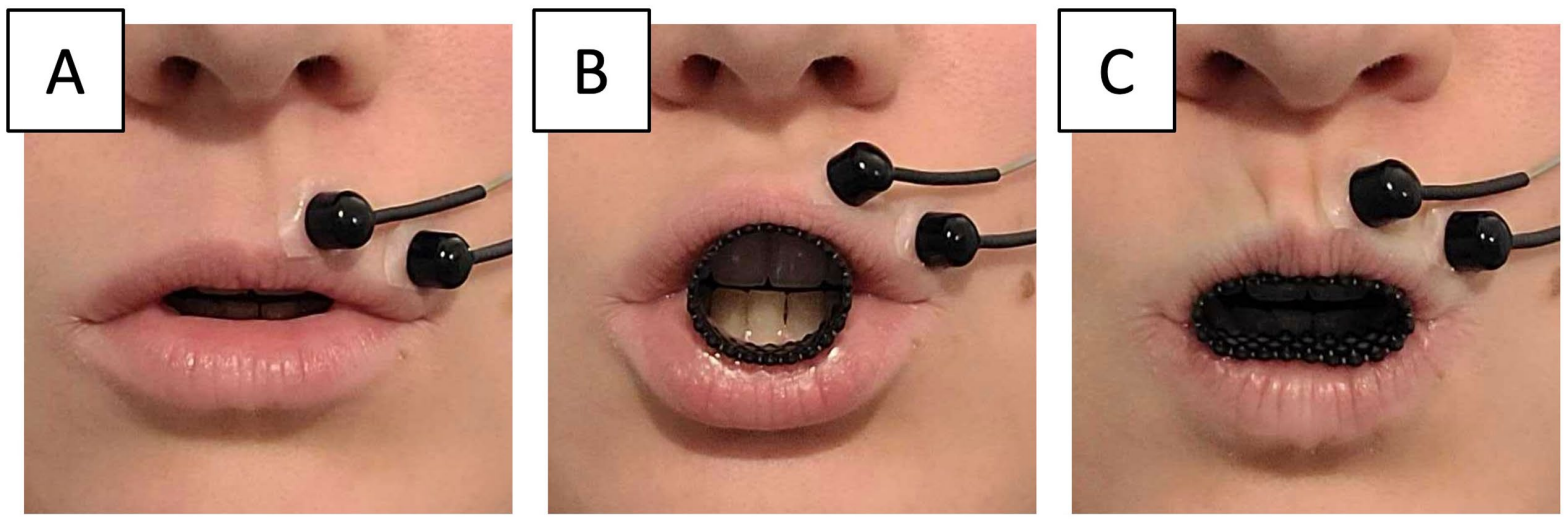
Production of vowel /ø/ with unconstrained lip movement **(A)** and with a deformable tube inserted between the lips **(B)**, shown in its deformed state **(C)**. Surface electromyography was used to characterize the activity of the orbicularis oris muscle.

In French, the contrast between /e/ and /ø/ is characterized acoustically by differences in the second formant (F2) and articulatorily, primarily by lip rounding and protrusion. Consequently, for naïve speakers (i.e., without specific knowledge of acoustic phonetics), perceiving their /ø/ productions as approaching /e/ was expected to elicit an enhancement of lip rounding. We therefore targeted lip rounding as the compensatory gesture of interest, and aimed to manipulate the effort involved in this gesture by using deformable lip tubes of different rigidities, placed between the lips during speech production. Unlike classical lip tube experiments (Savariaux et al., 1995; Beaudry et al., 2025), the lip tubes used in this experiment were deformable and compressible. They did not prevent nor restrict lip rounding kinematically, but instead, they exerted a mechanical resistance to the movement, thereby increasing the physical effort required to perform it (see Figure 2). Our purpose here was to test how speech adaptation to altered auditory feedback might be influenced by the amount of physical effort required to achieve acoustic compensation. More specifically, we examined whether increased physical effort would lead to reduced magnitude of acoustic compensation in response to comparable levels of auditory perturbation (H1a), and cause the acoustic compensation to reach possible saturation earlier, i.e., at lower levels of perturbation (H1b).

Two lip tubes of different rigidities, a very flexible lip tube (FLT), and a more rigid lip tube (RLT), were selected and compared in two consecutive sessions of the experiment. Both tubes were 1.5 cm in diameter and 2 cm in length. They were 3D-printed in polyurethane using a powder fusion process (HP Multi Jet Fusion), with two different wall thicknesses (respectively 1 and 2 millimeters), resulting in different mechanical stiffnesses. For a compression inducing a 6 mm reduction in diameter, FLT requires a force of 0.40 N whereas RLT requires a force of 2.25 N (see Supplementary Material 1 for more details). The tubes were placed between the lips so that, at rest, they touched the upper teeth and could remain in place without requiring any lip contraction. Each participant completed two sessions of the experimental procedure described above (6 blocks of speech production with unaltered auditory feedback, followed by 10 blocks of speech production under increasing formant perturbation), once with the very flexible lip tube (FLT) and once with the more rigid one (RLT), in a counterbalanced order across participants, and with a five-minute break between the two sessions.

Supplementary Material 2 provides details on the impact of these lip tubes on the production of vowel /ø/, even before any auditory feedback perturbation was applied. In summary, the lip tubes induced a small but significant upward shift of average F1 and F2 frequencies (by about +5%), as well as increased lip-muscle activity (by about +130%), compared to natural /ø/ productions with unconstrained articulation. This unintended effect can be regarded as a small bias introduced by our experimental paradigm. However, only negligible differences in formant frequencies (around 1% for both F1 and F2) were observed between the two lip-tube conditions, allowing for a valid relative comparison, and interpretation of their differences solely in terms of tube rigidity. Furthermore, the production of vowel /ø/ with the RLT already exhibited significantly greater lip muscle activity under unaltered auditory feedback, compared to the FLT condition, consistent with the expectation that participants would increase lip-rounding to reduce the interlip-area from the dimensions imposed by the tubes to the typical configuration for /ø/, which should require greater effort in the RLT condition.

#### 2.2.4 Reference productions

In addition to the two lip tube conditions, each participant completed 90 trials of the same picture-naming task, without any tube between the lips and without auditory feedback perturbation. These trials served to establish baseline values for the “natural” production of the vowel /ø/ and to assess the potential articulatory impact of the lip tube before any auditory-feedback manipulation.

Finally, in a last task of maximum voluntary contraction (MVC), participants were asked to press their lips as firmly as possible for 4 s against a fully rigid lip tube with the same diameter as the deformable tubes used in the experiment. Three repetitions were recorded, with a few seconds of rest between each.

### 2.3 Measurements

Speech was recorded using the integrated microphone of the headset (Audio-Technica BPHS1), connected to a sound card (RME Fireface 800), with 16-bit resolution and a sampling rate of 20 kHz. Since F2 is the acoustic variable intentionally modified by the auditory perturbation, and is critical for the phonological contrast between /ø/ and /e/, its measurement provides a reliable index of the magnitude of compensation in our study. However, because the articulatory adjustments underlying compensation affect the vocal tract as a whole, we expected not only F2 but the entire spectral envelope—particularly F1—to be modified in response to the perturbation. Therefore, both F1 and F2 frequencies were measured, rather than F2 alone. These values were extracted for each production using values detected by the Audapter software, within a time window from −100 ms to −50 ms before word offset. This interval was chosen to avoid the formant transitions associated with the voiced nasal [n], still observable near the syllable’s intensity peak, as well as the formant variations occurring at the end of the word.

Since compensation for F2-upshifted feedback during the production of the rounded vowel /ø/ was expected to rely mainly on increased lip-rounding movements, the electromyographic activity of the superior orbicularis oris muscle was recorded as a measure of the physical effort involved in this compensatory gesture. We hypothesized that lip-muscle activity would increase proportionally with the magnitude of acoustic compensation and, like F2 variations, would exhibit possible saturation beyond a certain level of auditory perturbation, reflecting the attainment of a functional effort threshold (H2).

To measure lip-muscle activity and test this hypothesis, a pair of surface electrodes (Biopac EL254S) was placed above the upper lip (see Figure 2), connected to a Biopac MP150 system and a National Instruments SCB-68 acquisition board, on 16 bits and at a rate of 2 kHz. Four participants had EMG recordings of insufficient quality and were excluded from the analysis, yielding a sample of 32 participants. The raw EMG signal was band-pass filtered between 20 and 450 Hz using a fourth-order zero-phase Butterworth filter to remove movement artifacts and high-frequency noise. Then, a zero-phase notch filter was applied to suppress power line interference at 50 Hz. The EMG root-mean-square value (RMS_EMG_) was calculated for each word over a 100 ms window centered on the peak muscle activation, which generally preceded the syllable’s peak intensity by a few milliseconds. For each participant, the average RMS of the EMG signal during the reference task—producing vowel /ø/ with unaltered auditory feedback—was used as a baseline to evaluate changes in muscle activity across the two lip tube conditions, and under altered auditory feedback.

In addition, the RMS of the EMG signal was calculated over a 100-ms window centered on the peak muscle activation during the MVC tasks. For each participant, the maximum RMS_MVC_ value was then taken as the highest value across the three repetitions.

The synchronous acquisition of both acoustic and EMG signals was performed using MATLAB software (R2022b), as well as the signal post-processing and the extraction of the three parameters of interest (F2, F1, RMS_EMG_) from these signals.

### 2.4 Statistical analyses

**Figure 3:**
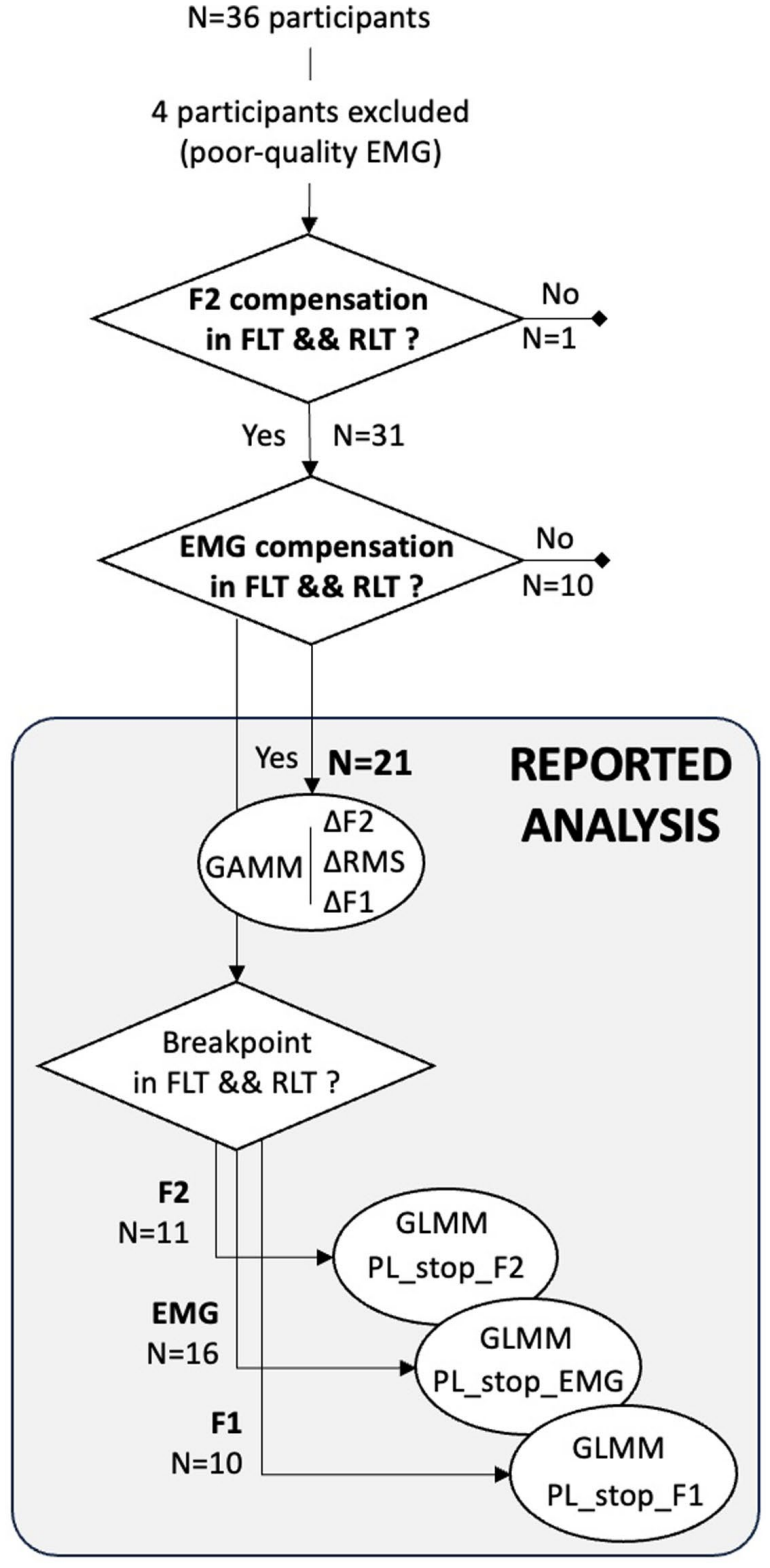
Flowchart illustrating the study analyses. Rounded shapes refer to each type of analysis and its dependent variable/s, and trapezoidal shapes refer to the participants included as well as the logic behind their selection.

Statistical analyses were conducted using R software (R Core Team, 2022). Distinct statistical analyses were performed to address different issues. The flowchart shown in Figure 3 summarizes these analyses, the participants included in each of them, and the corresponding selection logic.

We assessed compensation ability of each participant (*N* = 32) in both FLT and RLT conditions by testing whether they significantly decreased the F2 of their vowel /ø/ in response to a moderately altered auditory feedback—35% F2 upshift—similar to perturbation levels used in previous studies (Villacorta et al., 2007; Tourville et al., 2008; Bourguignon et al., 2014; van Den Bunt et al., 2017). To this end, F2 values were first normalized relative to the participant’s average F2 with unaltered feedback in the same condition, yielding a percent variation ratio ΔF2 (%). One-sample *t*-tests, implemented via intercept-only linear models (*lm* function in R), were then used to determine, for each participant and condition, whether ΔF2 at 35% perturbation significantly differed from zero. One subject did not compensate for the formant perturbation in any condition, and was therefore excluded from further analysis.

Then, for each remaining participant (*N* = 31) and each condition, we assessed to what extent the acoustic compensation involved increased lip rounding, by testing whether lip muscle activity increased significantly between 0 and 35% of auditory perturbation. Ten of the 31 participants showed a significant increase in lip muscle activity in only one of the two lip tube conditions (four in FLT condition; six in the RLT condition), suggesting that their acoustic compensation of the F2-upshifted feedback did not consistently rely on the expected lip-rounding strategy targeted by our experimental design. This implies that the mechanical resistance induced by the flexible lip tubes—the main experimental manipulation of the present study—either did not induce a compensation strategy based on lips movements, or did not induce a consistent compensation strategy across conditions, preventing us from conducting a comparison between conditions. The final analysis therefore relied on 21 participants who behaved in accordance with the experimental design, that is, who compensated in F2 and recruited their lips to achieve this acoustic compensation in both conditions. For completeness, the behavior of the 10 remaining participants who compensated in F2 but without systematically recruiting their lips, is reported in the Supplementary Material 4.

Based on this group of “lip compensators” (*N* = 21), we tested whether the rigidity of the lip tube affected:

- The degree of F2 compensation (H1a). This analysis was based on generalized additive mixed models (GAMMs) implemented in the R package *mgcv*. In these models, perturbation level was treated as a continuous predictor and modeled using condition-specific smooth functions, allowing for potentially non-linear relationships between perturbation magnitude and the compensatory responses. Subject-specific variability was accounted for by including a random intercept for participant as well as participant-specific smooths of perturbation (factor-smooth interactions), thereby allowing non-linear participant-level deviations from the population-level smooth. Model estimation was performed using restricted maximum likelihood (REML). For model comparison using likelihood ratio tests, models were refitted using maximum likelihood (ML), following standard recommendations (Wieling, 2018). The primary effect of tube rigidity was assessed by estimating the difference between the condition-specific smooths (TLS vs. TLR) across the perturbation range. Statistical significance of these differences was evaluated using simultaneous confidence intervals derived from the fitted GAMMs.
- The level of perturbation at which F2 compensation began to saturate (H1b). This analysis first relied on the characterization of non-linear changes in vowel articulation across perturbation levels. For each participant and each lip-tube condition, we applied a piecewise linear regression to the relative variations in F2 (using two segments), in order to detect a possible saturation effect and, when present, to estimate the perturbation level at which it occurred (PL_stop_F2). F2 was considered to exhibit a saturation effect when the following criteria were met: (1) a significant difference between the slopes of the two segments, indicating that a two-segment regression accounted better for the variation in F2 values than a single-slope regression; and (2) the slope at higher perturbation levels was either not significantly different from zero, or significantly positive. The breakpoints between segments were estimated using a grid-search procedure, constrained to include a minimum of five data points per segment. The slopes of the two different segments as well as their pairwise differences, were statistically evaluated to assess changes in compensatory responses.

Based on this preliminary non-linearity analysis, we further examined in the 11 participants showing F2 saturation in both lip-tube conditions whether saturation of the F2 compensation occurred at lower perturbation levels for the more rigid tube (RLT) compared to the more flexible one (FLT) (H1b). This was tested using a linear mixed-effects model, with tube rigidity as a fixed effect (2 levels: FLT; RLT), and participant as a random effect (PL_stop_F2 ~ tube rigidity + 1|participant).

Although F2 was the main acoustic parameter of interest for characterizing compensation, as it was the parameter directly perturbed in the auditory feedback, similar and complementary analyses were also performed on F1, which was also expected to vary with changes in vowel articulation and could therefore provide additional support for hypotheses H1a and H1b, or complementary information.

To better interpret the results of the preceding analyses and determine to what extent an earlier saturation in F2 compensation could be related to lip muscle activity reaching a physiological maximum more rapidly (H2), we conducted the following complementary analyses:

- A GAMM analysis similar to that described previously for testing H1a on F2 variations was performed to assess the influence of tube rigidity on the magnitude of variations in lip muscle activity (RMS_EMG_).
- A similar analysis of non-linear changes in lip muscle activity across perturbation levels, as described previously for testing H1b on F2 variations, was conducted to detect a possible saturation effect in EMG variations and to estimate the perturbation level at which it occurred (PL_stop_EMG). In the case of EMG, piecewise linear regression was applied to the relative variations in RMS_EMG_ using three segments. This modeling choice was motivated by the observation of a third phase of decreasing activity at the highest perturbation levels, in addition to the expected increase and plateau phases. Variations in lip muscle activity were considered to exhibit a saturation effect when the following criteria were met: (1) a significant difference between the slopes of adjacent segments; (2) in adjacent segments, the “left” one—corresponding to lower perturbation levels, presented a significantly positive slope, whereas that of the “right” one—corresponding to higher perturbation levels, was either not significantly different from zero, or significantly negative.
- Similarly to the analysis conducted for H1b on F2 variations, we further examined, in the 16 participants who exhibited EMG saturation in both conditions, whether this saturation occurred at lower perturbation levels for the more rigid tube (RLT) than for the more flexible tube (FLT). This was tested using again a linear mixed-effects model, with tube rigidity as a fixed effect (2 levels: FLT; RLT), and participant as a random effect (PL_stop_EMG ~ tube rigidity + 1|participant).
- Finally, we examined the coincidence between the perturbation levels at which saturation occurred in F2 and RMS_EMG_ (PL_stop_F2 and PL_stop_EMG) across all cases showing a co-occurring saturation effect in both F2 and RMS_EMG variations (Participant × Condition; *N* = 24 cases). We computed a difference score between the two breakpoints (PL_stop_F2 − PL_stop_EMG) and analyzed it using a linear mixed-effects model with tube rigidity as a fixed effect (FLT; RLT), and participant as a random effect (PL_stop_F2-PL_stop_EMG ~ tube rigidity + 1|participant), in order to account for the repeated-measures structure of the data. The primary question of interest was whether the mean difference between breakpoints differed from zero, which was assessed by testing the intercept of the model.
- A similar analysis was run to examine the coincidence between the perturbation levels at which saturation occurred in F1 and RMS_EMG_ (PL_stop_F1 and PL_stop_EMG) across all cases showing a co-occurring saturation effect in both F1 and RMS_EMG variations (Participant × Condition; *N* = 25 cases).

## 3 Results

### 3.1 Effect of lip tube rigidity on the magnitude of F2 acoustic compensation (H1a)

**Figure 4:**
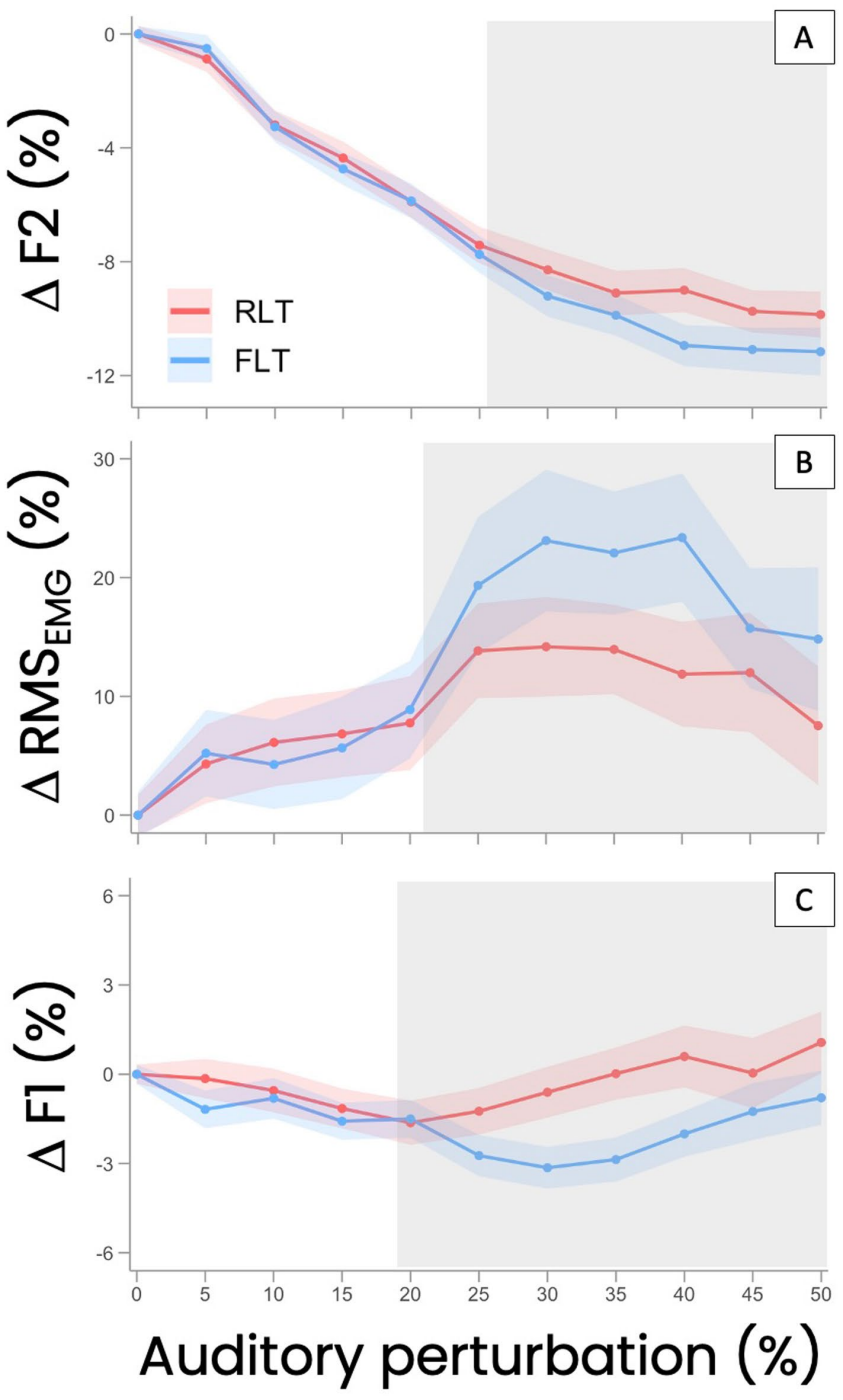
Relative variations in the frequency of the second formant (ΔF2) **(A)**, in lip muscle activity (ΔRMS_EMG_) **(B)** and in the frequency of the first formannt (ΔF1) **(C)**, during the production of vowel /ø/ under progressively increased auditory feedback perturbation (0 to 50% F2 upshift) relative to the values of these parameters under unaltered auditory feedback, with either a very flexible (FLT) or a more rigid (RLT) lip tube inserted between the lips. Thick lines and shaded ribbons represent the mean and 95% confidence intervals across participants (*N* = 21) at each perturbation level. Gray areas highlight the perturbation levels at which significant differences were observed between the two lip tube conditions.

The degree of F2 compensation (ΔF2, expressed as a percentage change relative to the average F2 value under unaltered auditory feedback) increased with the level of perturbation, in different ways across the two lip-tube conditions (see Figure 4A). A Generalized Additive Mixed Model including condition-specific smooths of perturbation provided a significantly better fit than a model assuming a common smooth across conditions (likelihood ratio test: df = 4.22, *χ*^2^ = 467.36, *p* < 0.0001***), indicating a significant tube rigidity * perturbation level interaction. Inspection of the smooth difference between conditions revealed that compensation did not significantly differ between FLT and RLT at low perturbation levels (0–25%), as the simultaneous confidence intervals of the smooth difference included zero across this range. From approximately 26% perturbation onward, compensation became significantly greater in the FLT condition than in the RLT condition (simultaneous confidence intervals excluding zero), and this difference remained significant up to the highest perturbation level tested (50%). The magnitude of the between-condition difference increased progressively with perturbation level, reaching a maximum difference of approximately 1.55% around 42–44% perturbation before slightly decreasing at the highest levels while remaining statistically significant.

### 3.2 Effect of lip tube rigidity on the non-linear dynamics of F2 acoustic compensation across increasing levels of auditory perturbation (H1b)

**Figure 5:**
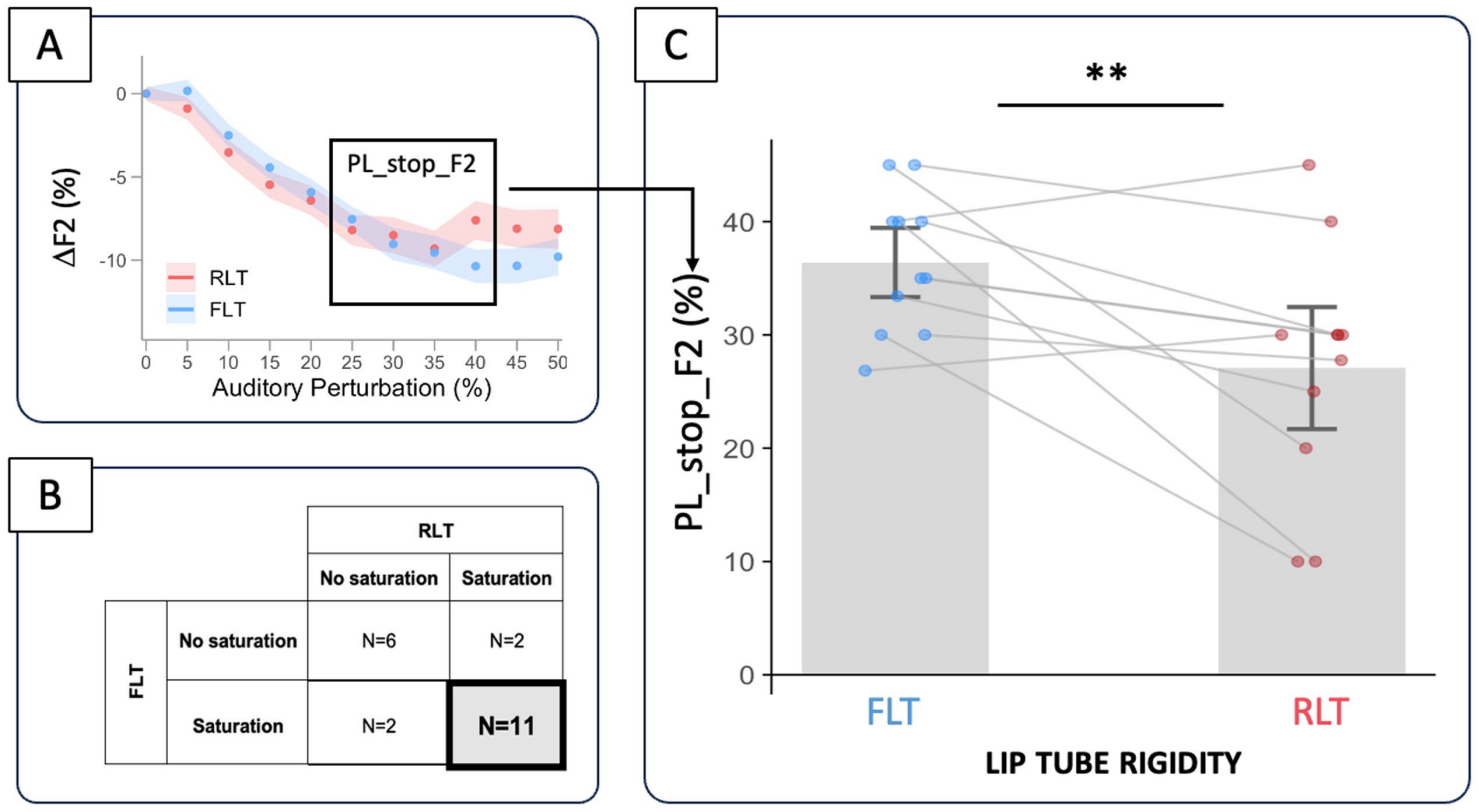
Comparison of PL_stop_F2—the level of auditory perturbation at which F2 compensation (ΔF2) begins to saturate **(A)**—between the FLT and RLT conditions **(C)**, for the 11 participants (out of 21) who exhibited ΔF2 saturation in both conditions **(B)**.

The variation in F2 showed a non-linear relationship with the level of auditory perturbation (see Figure 4A). The two-slope regression analysis revealed a slowdown, saturation, or even a reduction in compensation at higher perturbation levels. In the FLT condition, this pattern was observed in 13 of the 21 participants, beginning on average at 34.6 ± 8.1% perturbation. In the RLT condition, a similar effect was found in 13 participants, beginning on average at 29.0 ± 11.1% perturbation. Eleven participants exhibited this saturation trend in both conditions (see Figure 5B). For them, the perturbation level at which saturation started (PL_stop_F2) tended to be lower for the very rigid lip tube (RLT) compared to the more flexible one (FLT) (−9.3 ± 3.2%, *p* = 0.004**) (see Figures 5A,C).

### 3.3 Relationship with variations in lip muscle activity (H2)

For the 21 participants included in these analyses—participants whose acoustic compensation was accompanied by a significant increase in lip muscle activity, suggesting enhanced lip rounding—variations in lip muscle activity (ΔRMS_EMG_) revealed a nonlinear pattern: an initial increase followed by a plateau and a subsequent decline at higher perturbation levels, in both tube conditions (see Figure 4B). A Generalized Additive Mixed Model allowing condition-specific smooths of perturbation provided a significantly better fit than a model assuming a common smooth across conditions (likelihood ratio test: df = 3.84, *χ*^2^ = 22,385, *p* < 0.0001***), indicating a significant tube rigidity * perturbation level interaction. At low perturbation levels (0–21%), the difference between conditions was not statistically significant, as the simultaneous confidence intervals of the smooth difference included zero across this range. From approximately 21% perturbation onward, lip muscle activity became significantly greater in the RLT condition than in the FLT condition (simultaneous confidence intervals excluding zero), and this difference remained significant up to the highest perturbation level tested (50%). The magnitude of the between-condition difference increased progressively with perturbation level, reaching a maximum difference of approximately 10–10.5% around 34–36% perturbation. At higher perturbation levels, lip muscle activity decreased in both conditions, but the difference between conditions remained statistically significant.

**Figure 6:**
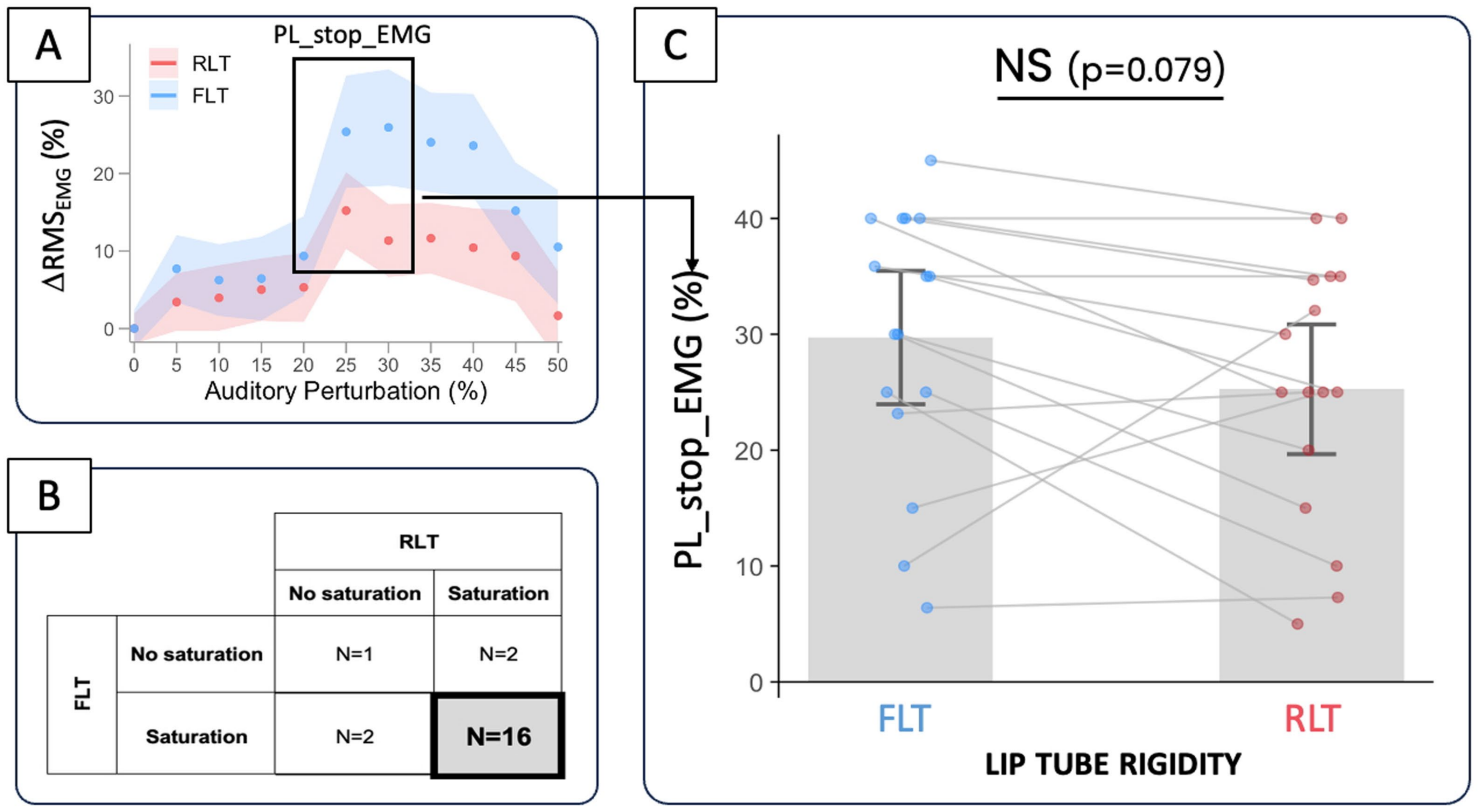
Comparison of PL_stop_EMG—the level of auditory perturbation at which variation in lip muscle activity (ΔRMS_EMG_) begins to saturate **(A)**—between the FLT and RLT conditions **(C)**, for the 16 participants (out of 21) who exhibited saturation trend in both conditions **(B)**.

Individually, this saturation trend in the variations of lip muscle activity was observed in 18 participants in the FLT condition, with the saturation effect beginning on average at 30.0 ± 11.4% perturbation. In the RLT condition, it was also observed in 18 participants, with saturation beginning on average at 27.4 ± 12.2%. Among the 16 participants who exhibited saturation of lip muscle activity in both lip-tube conditions (see Figure 6B), the perturbation level at which saturation began tended to be lower in the RLT condition than in the FLT condition (see Figures 6A,C), although this difference did not reach statistical significance (−4.5 ± 2.5%, *p* = 0.079).

**Figure 7:**
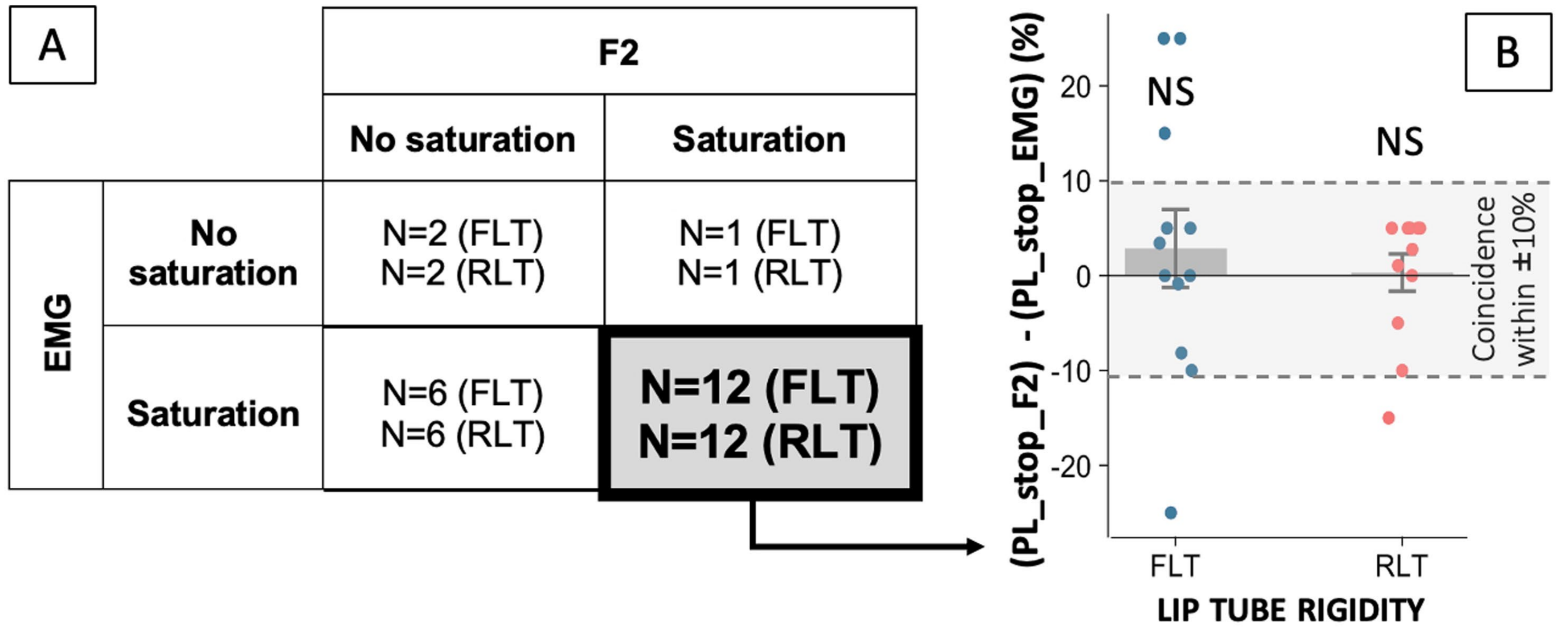
Distance between the perturbation levels at which F2 and lip muscle activity start to saturate (PL_stop_F2 and PL_stop_EMG) **(B)** for participants showing a co-occurring saturation effect in both variables (*N* = 24) **(A)**. The dashed lines indicate the region in which both breakpoints coincide within ±10%.

Similar patterns of variations in F2 and lip muscle activity were observed in 67% of the cases (out of 21 participants × 2 lip-tube conditions), either because no saturation was observed in either variable (4 cases), or because both variables exhibited saturation (24 cases; see Figure 7A). Among these 24 concomitant saturation cases, the perturbation levels at which saturation occurred in both variables, PL_stop_F2 and PL_stop_EMG coincided within ±10% in 19 instances (see dashed lines of Figure 7B). The average distance between the two breakpoints did not differ significantly from zero (1.6 ± 2.2%, *p* = 0.48), similarly across the two lip-tube conditions [*F*(1, 22) = 0.31, *p* = 0.58]. Furthermore, in the remaining 33% of cases, saturation was observed in F2 but not in EMG, or vice versa (see Figure 7A).

### 3.4 F1 variations and articulatory strategies

The preceding results, showing that variations in F2 and its saturation at high perturbation levels cannot be strictly predicted from lip muscle activity, suggest that in some participants, the compensation for the auditory perturbation also involves a contribution from an alternative articulatory movement. Although lip rounding was expected to be the primary articulatory strategy for lowering F2 in the rounded vowel /ø/—especially in contrast with the spread vowel /e/—a backward tongue movement could also lower F2. While our study does not include direct articulatory measurements of lip, jaw, or tongue motions, variations in F1—and more precisely, its covariation with F2—provide indirect but quantitative insight into the articulatory adjustments that may underlie the observed acoustic compensation. Indeed, according to Fant’s nomograms (Fant, 1970), both lip rounding and tongue backing, in the vocal tract configuration of the vowel /ø/ with the lip tube inserted between the lips, lower F2 but have opposite effects on F1: lip rounding decreases F1, whereas tongue backing increases it. No other articulatory movement but tongue backing would account for a simultaneous rise in F1 and decrease in F2 (see Supplementary Material 5 for acoustic simulations supporting this assertion).

Across the whole group of “lip compensators” (*N* = 21), F1 also exhibited non-linear variations, particularly in the RLT condition, with a general trend toward a slight decrease at low levels of auditory perturbation (from 5 to 20%), followed by a breakpoint where F1 began to increase, returning to its average value under unaltered feedback for the FLT condition, or even exceeding it for the RLT condition (see Figure 4C). A Generalized Additive Mixed Model allowing condition-specific smooths of perturbation provided a significantly better fit than a model assuming a common smooth across conditions (likelihood ratio test: df = 6.86, *χ*^2^ = 1288.7, *p* < 0.0001***), indicating a significant tube rigidity × perturbation level interaction. At low perturbation levels (0–18%), the difference between the two lip-tube conditions was not statistically significant, as the simultaneous confidence intervals of the smooth difference included zero across this range. From approximately 19% perturbation onward, ΔF1 became significantly greater in the TLS condition than in the TLR condition (simultaneous confidence intervals excluding zero), and this difference remained significant up to the highest perturbation level tested (50%). The magnitude of the between-condition difference increased progressively with perturbation level, reaching a maximum difference of approximately 2.6% around 34–35% perturbation, before gradually decreasing at higher perturbation levels, while remaining significantly positive. This suggests that the contribution of the alternative articulatory strategy—probably tongue backing—was greater in the RLT condition and increased when the effort required at the lips to compensate for large F2 upshifts reached a certain level.

**Figure 8:**
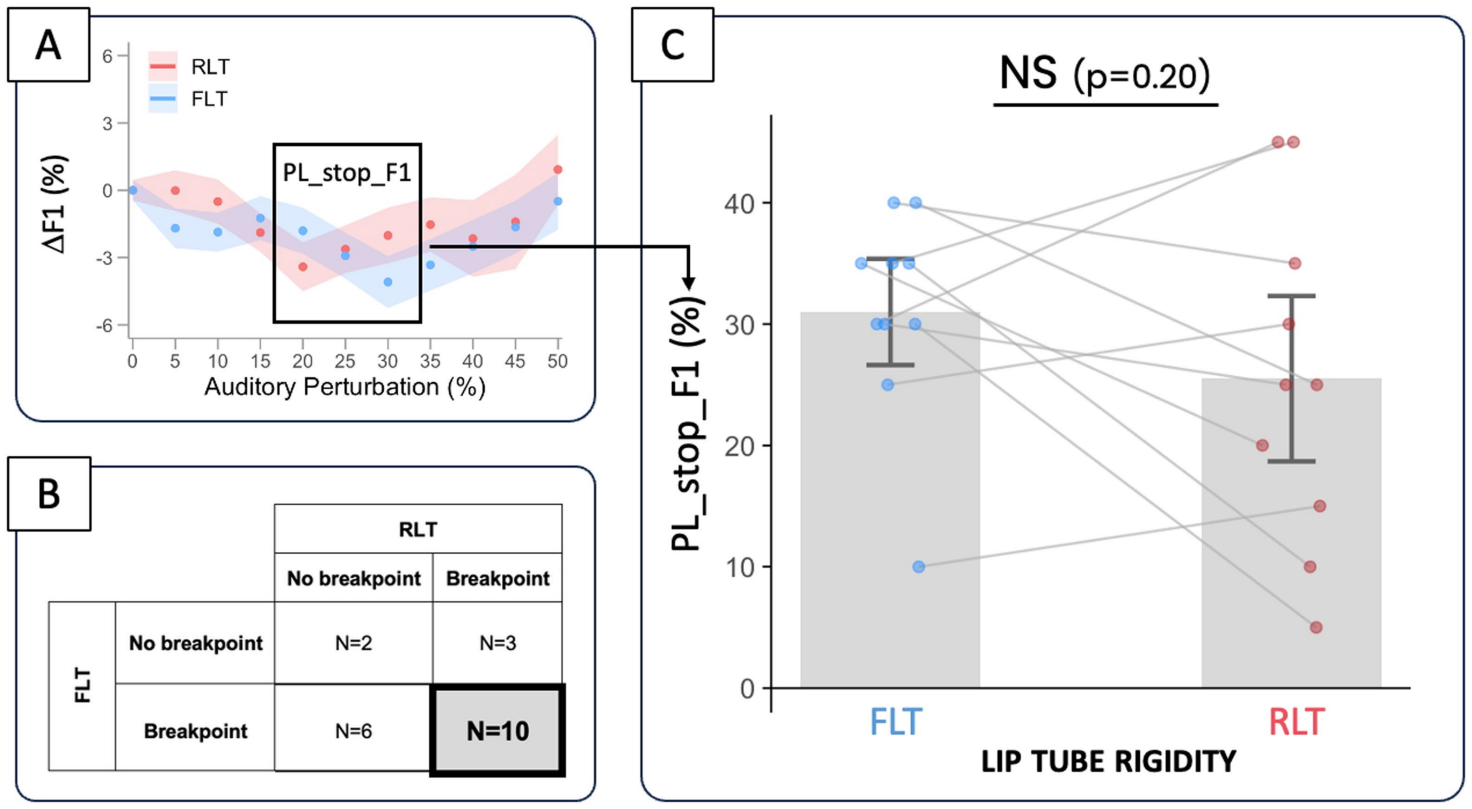
Comparison of PL_stop_F1—the level of auditory perturbation at which variations in F1 (ΔF1) show a significant breakpoint **(A)**—between the FLT and RLT conditions **(C)**, for the 10 participants (out of 21) who exhibited such a breakpoint in both conditions **(B)**.

At individual level, such a trend was observed in 16 participants in the FLT condition, with a change in direction of the F1 variation situated around 31.0 ± 9.8% of perturbation, on average. In the RLT condition, it was also observed in 13 participants, with a breakpoint around perturbation level of 23.8 ± 13.2%. For the 10 participants who exhibited a significant breakpoint in their F1 variation in both lip-tube conditions (see Figure 8B), the perturbation level at which this change in compensatory behavior occurs tended to be lower in RLT compared to FLT (see Figures 8A,C), but this tendency was not statistically significant (−5.5 ± 4.3%, *p* = 0.20).

**Figure 9:**
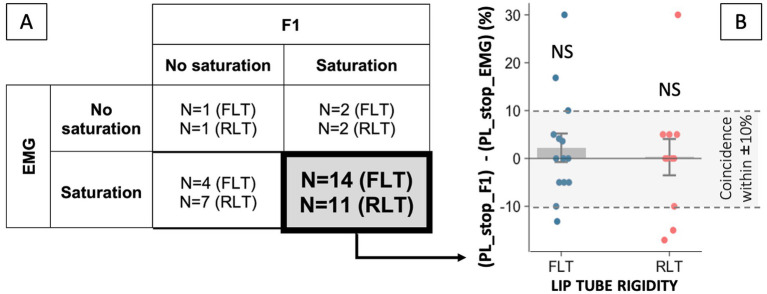
Distance between the perturbation levels at which F1 and lip muscle activity start to saturate (PL_stop_F1 and PL_stop_EMG) **(B)** for participants showing a co-occurring saturation effect in both variables (*N* = 25) **(A)**. The dashed lines indicate the region in which both breakpoints coincide within ±10%.

Similar patterns of variations in F1 and lip muscle activity were observed in 64% of the cases (out of 21 participants × 2 lip-tube conditions), either because no saturation was observed in either variable (2 cases), or because both variables exhibited saturation (25 cases; see Figure 9A). Among these 25 concomitant saturation cases, the perturbation levels at which saturation occurred in both variables, PL_stop_F1 and PL_stop_EMG coincided within ±10% in 20 instances (see dashed lines of Figure 9B). The average distance between the two breakpoints did not differ significantly from zero (1.4 ± 2.4%, *p* = 0.57), similarly across the two lip-tube conditions [*F*(1, 23) = 0.17, *p* = 0.68]. Furthermore, in the remaining 36% of cases, saturation was observed in F1 but not in EMG, or vice versa.

Table 1 provides a summary of the results of the different analyses described above.

**Table 1:** Summary of the statistical analyses results.

Question	Tested effect	Model	Variable	Sample	Result	Details	Direction
Effect of tube rigidity on the degree of compensation	Tube rigidity × perturbation level	GAMM	ΔF2	21	Significant	[26–50%]	FLT > RLT
			ΔRMS_EMG_	21	Significant	[21–50%]	FLT < RLT
			ΔF1	21	Significant	[19–50%]	FLT > RLT
Effect of tube rigidity on the saturation point	Tube rigidity	GLMM	PL_stop_F2	11	Significant	−9.3 ± 3.2%, *p* = 0.004	FLT > RLT
			PL_stop_EMG	16	n.s.	−4.5 ± 2.5%, *p* = 0.079	—
			PL_stop_F1	10	n.s.	−5.5 ± 4.3%, *p* = 0.20	—
Breakpoint coincidence	Mean difference from 0	GLMM	PL_stop_F2 PL_stop_EMG	12 in FLT 12 in RLT	n.s.	1.6 ± 2.2%, *p* = 0.48	—
			PL_stop_F1 PL_stop_EMG	14 in FLT 11 in RLT	n.s.	1.4 ± 2.4%, *p* = 0.57	—

## 4 Discussion

### 4.1 Influence of physical effort on speech auditory-motor adaptation

Thanks to an original experimental paradigm using deformable lip tubes, our study tested how an increased lip tube rigidity, thereby increasing the physical effort needed for lip rounding without preventing the gesture, significantly influenced the adaptation to F2-upshifted auditory feedback during the production of the rounded vowel /ø/. The lip tubes can be considered as introducing a mild articulatory perturbation, since they elicited significant increases in the first two formant frequencies and in lip muscle activity during the production of /ø/ under unaltered auditory feedback. This likely reflects the fact that the tube diameter exceeded the typical inter-lip area for /ø/ in most participants. Because of this methodological bias, the compensatory strategies observed here may not be entirely comparable to those that would occur with unconstrained lip articulation. Nevertheless, the primary objective of this paradigm was to manipulate the level of articulatory effort required for lip-rounding, in order to compare speech adaptation to altered auditory feedback under otherwise similar production conditions, differing only in the biomechanical effort needed to achieve the compensatory gesture. In this respect, since the two lip-tube conditions produced only negligible acoustic differences in the absence of auditory perturbation, this supports the validity of the paradigm and the relevance of the results discussed below.

Consistent with previous studies on pitch- (Liu and Larson, 2007; Liu et al., 2010; Scheerer et al., 2013) or F1-shifted auditory feedback (MacDonald et al., 2010; Katseff et al., 2012), our results show that compensation for F2-shifted feedback during /ø/ production also reaches a plateau at high levels of auditory perturbation. This saturation was observed in most of the cases (62%), started from about 32% of perturbation, and reached an average magnitude of compensation of 10%, which is similar in magnitude to previous findings (Trudeau-Fisette et al., 2017). Most importantly, and in closer relation to our research question, increased lip tube rigidity reduced F2 compensation at higher perturbation levels (above 26%)—by approximately 1.5%—confirming our first hypothesis (H1a), and triggered the saturation of F2 compensation earlier, i.e., at lower perturbation levels, by 9.3% on average—which is also consistent with our second hypothesis (H1b). However, this second finding is based only on a relatively small subset of participants (*N* = 11) who showed saturation in both lip-tube conditions. It is very likely that all participants ultimately reach a saturation point, but for some, this may occur at perturbation levels higher than those tested in the present study (0–50%). To confirm and generalize these results, future research should therefore consider extending the range of auditory perturbation levels.

Furthermore, by examining EMG data, our study sheds light on how lip muscle activity supports speech compensation in the acoustic domain. First, contrary to our expectations, only 21 of the 31 “acoustic compensators” showed a significant increase in lip muscle activity under altered auditory feedback. For the others, compensation for F2-upshifted feedback during /ø/ production relied on another articulatory movement very likely to involve tongue backing. For the 21 participants who recruited their lips under altered auditory feedback conditions, a general trend was observed, similar to the F2 variations, consisting of an initial increase in lip muscle activity at low perturbation levels, followed in most cases (86%) by a plateau or even a decrease at higher levels. In the RLT condition, there was a slowdown of about 10% in the increase of lip muscle activity at perturbation levels between 30 and 40%, where activity saturates—which further confirmed our hypothesis (H1a). There was also a tendency for saturation to occur earlier, i.e., at lower levels of perturbation—by 3.8% on average—in participants who exhibited saturation under both lip tube conditions—supporting the hypothesis (H1b). However, this trend did not reach statistical significance (*p* = 0.078) and was observed only in a limited subset (*N* = 10) among participants who showed EMG saturation in both lip-tube conditions. To confirm and generalize this pattern, future research will need again to extend the range of auditory perturbation levels, so that the saturation point can be observed in all participants and its onset compared across different levels of lip-tube rigidity. Further supporting the similar trend observed in the variations of both F2 and lip muscle activity, the perturbation levels at which both variables began to saturate (PL_stop_F2; PL_stop_EMG) were found to coincide within ±10% in about 80% of the cases—with the mean difference between these breakpoints not significantly differing from zero across participants.

Examining the absolute level of lip muscle activity provides additional insight to better understand saturation phenomena in the variation of both F2 and lip muscle activity. Indeed, under unaltered auditory feedback, producing vowel /ø/ with the more rigid lip tube (RLT) requires greater muscle engagement than with a more flexible lip tube (FLT) (see Supplementary Material 2). This offset toward higher EMG activity in the RLT condition is still observed throughout the lower perturbation levels, corresponding to the phase where lip muscle activity increases. In contrast, during the saturation phase at higher levels of perturbation, no significant difference in lip muscle activity remains between the two lip tube conditions (see Supplementary Material 3). Since the saturation level of lip muscle activity corresponds to only about 56.4% of the maximum voluntary contraction, far from the maximum possible contraction of this muscle, this ceiling effect does not seem to reflect a physiological or biomechanical limit. It probably rather corresponds to the upper boundary of a usual or comfortable range of muscle activation in speech production.

Altogether, these results point out the role of motor effort in shaping compensatory responses to auditory perturbations. The use of lip tubes of different rigidity enabled us to provide evidence for the existence of a maximum acceptable effort, beyond which participants appear unwilling or unable to go, thereby restricting the magnitude of the compensation and rendering it incomplete. So far, explanations for incomplete compensation to altered auditory feedback, and for variations in the magnitude of compensation, have focused on individual differences in auditory acuity and on the width of the acoustic space defining the target speech-sound category, such that bringing the auditory feedback back within an “acceptable” acoustic region may be sufficient (Villacorta et al., 2007; Mitsuya et al., 2011). It has also been proposed that compensation reflects a trade-off between restoring the auditory target and preserving somatosensory compatibility with the intended phonological category (Patri et al., 2018). On top of these limitations, our results suggest that motor cost is also an important constraint on speech sensorimotor adaptation, and more generally on the regulation of speech production.

### 4.2 Selection of a compensatory strategy

Using deformable lip tubes in this experiment, we expected participants to produce the rounded vowel /ø/ with increased lip rounding when their auditory feedback was F2-upshifted toward the perception of a spread vowel /e/. Consistent with this prediction, 21 of the 31 compensating participants increased lip muscle activity in both conditions to achieve acoustic compensation. However, F2 and EMG variations were not fully correlated across the entire range of perturbation levels. At higher perturbation levels in particular, F2 sometimes continued to decrease while lip muscle activity reached saturation, or conversely, F2 saturated while lip muscle activity declined. Moreover, 10 participants were able, in one of the two lip-tube conditions, to lower their F2 in response to the increasingly upshifted feedback without increasing lip muscle recruitment. These observations therefore suggest that, in some cases, compensation for the F2-upshifted feedback relied on an alternative articulatory strategy, very likely to involve tongue backing, consistent with previous findings on the production of the rounded vowel /u/ with a rigid (non-deformable) lip tube (Savariaux et al., 1995; Guenther et al., 1998). Future research might include articulatory measurements to confirm the involvement of tongue backing as the alternative articulatory strategy, which is, *a priori*, the only movement expected to produce a simultaneous increase in F1 and decrease in F2 (Fant, 1970; see also stimulations in Supplementary Material 5).

In any case, the existence of an alternative compensatory strategy—regardless of its precise articulatory nature, alongside the expected strategy based on lip rounding, certainly explains a large part of the inter-individual variability observed in our data (see in particular Section 3.3). Choosing a different speech task in our experimental paradigm might have reduced this variability and yielded clearer, more systematic compensatory behaviors across participants. However, our choice also allowed us to go beyond simply observing and quantifying how articulatory effort limits the magnitude of the compensation for an auditory perturbation once a motor plan (i.e., predominantly lip rounding) has been selected to achieve the required speech task. It enabled us to address, in line with the experimental work of Cos et al. (2011) in arm motor control (e.g., Schweighofer et al., 2015), the question of how motor effort may influence the selection of the motor plan itself (i.e., lip rounding vs. alternative strategy—probably tongue backing). Indeed, among the 21 participants who increased lip muscle activity in both lip-tube conditions (21 × 2 = 42 sessions), we observed 25 sessions in which the increase of F1 (indicating a predominant contribution of the alternative articulatory strategy of compensation (probably tongue backing) at high perturbation levels) was synchronized with the saturation of the lip muscle activity (see Figure 9). This suggests that in these sessions, participants, faced with an undesirable increase in the effort required to further enhance lip rounding, shifted their compensatory strategy toward tongue backing, allowing them to continue lowering F2, albeit at the cost of increasing F1. Obviously, to draw fully conclusive inferences, future work should evaluate the effort associated with tongue-backing movements and assess how this effort is perceived by participants, relative to that required for increased lips rounding.

An alternative explanation for the inter-individual variability in our data, and for the change in compensatory behavior at higher perturbation levels, may lie in differences in participants’ awareness of the perturbation, or in the perturbation becoming consciously detectable beyond a certain level. In previous studies applying gradual formant-shifted perturbations to the auditory feedback, typically reaching levels of about 35%, most of the participants did not consciously perceive that their auditory feedback had been altered, nor that they had modified their articulation to compensate for the perturbation (Kim and Max, 2021). In such studies, auditory-motor adaptation of speech therefore predominantly relies on an *implicit* mechanism that operates outside of awareness (McDougle et al., 2015; Taylor et al., 2014). In our study, which explored similar ranges of perturbation levels as previous work—but also higher ones, from 35% up to 50%—some participants may have become aware of the auditory perturbation or the compensatory adjustments at some point. This awareness could arise either from alterations in vocal timbre induced by Audapter’s transformation of the spectral envelope, or from a sense of incongruency between auditory and somatosensory feedbacks. In sensorimotor adaptation studies of arm movements (both visuomotor and force-field paradigms), being aware of the perturbation does not prevent compensation, but instead gives rise to *explicit* adaptations that can involve deliberate re-aiming strategies (Huberdeau et al., 2015). In line with this idea, the switch from a lip-rounding strategy to a tongue-backing strategy observed in our study at higher auditory perturbation levels could be interpreted as a deliberate re-aiming response once the perturbation is detected and the adaptation becomes explicit. However, several findings indicate that insights from visuomotor adaptation cannot be directly transferred to speech auditory-motor adaptation, as the latter appears to be predominantly implicit and may lack an explicit component. Indeed, previous studies have shown that speech compensation tends to be attenuated when participants are informed about, and become aware of the auditory perturbation (Munhall et al., 2009; Kim and Max, 2021). Furthermore, it has been shown that reach visuomotor adaptation—specifically its explicit component—is reduced when performed concurrently with speech auditory-motor adaptation, whereas the reverse interference is absent. This asymmetry has been interpreted as further evidence that speech auditory-motor adaptation may lack an explicit component (Lametti et al., 2020). Hence, although we cannot rule out the possibility that explicit processes contributed to the changes in compensatory behavior observed in our study at higher levels of auditory perturbation, any such contribution was likely limited.

## 5 Conclusion

This study provides evidence that the physical effort required to perform compensatory gestures influences the extent and dynamics of speech sensorimotor adaptation, and possibly also the choice for the articulatory movement adapted to achieve compensation. By experimentally manipulating the mechanical resistance of lip rounding through deformable lip tubes, we showed that increased articulatory effort leads to smaller and earlier-saturating compensatory responses to altered auditory feedback. The concomitant nonlinear variations in lip-muscle activity and in acoustic output suggest that speech adaptation is constrained by both sensory feedback mechanisms and the biomechanical costs associated with corrective movements.

Beyond its methodological contribution, these findings offer new insight into the sources of inter-individual variability in speech adaptation. Differences in perceived or actual articulatory effort may partly explain why some speakers exhibit weaker or incomplete compensation when faced with auditory perturbations. This perspective may be particularly relevant for clinical populations, such as individuals with Parkinson’s disease or stuttering, who often experience increased or dysregulated effort in speech production. Understanding how effort shapes adaptive control may thus help interpret reduced adaptation in these disorders and guide the design of therapeutic interventions that target both sensorimotor learning and effort regulation in speech.

## Data Availability

The raw data supporting the conclusions of this article will be made available by the authors, without undue reservation.
