# Supplementary material for: Articulatory effort modulates speech adaptation to auditory perturbations
Source: Front Hum Neurosci. 2026 May 22;20:1756554. doi: 10.3389/fnhum.2026.1756554 (PMC13236938; doi:10.3389/fnhum.2026.1756554)
Supplement: Supplementary file 1 [file Data_Sheet_1.PDF]

Supplementary Material

Supplementary Material 1 - Mechanical properties of the two deformable lip tubes

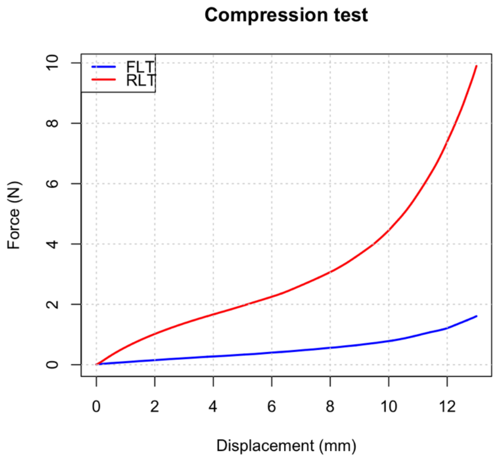

Supplementary Figure S1. Force–displacement relationship obtained during the compression test of the very flexible (FLT) or more rigid (RLT) lip tubes.

Supplementary Material 2 - Evaluation of the experimental paradigm: Impact of the deformable lip tubes on vowel production under unaltered auditory feedback

Across the entire group (N=36 for acoustic measures; N=32 for EMG measures), we tested how the lip tubes affected the production of vowel /ø/. More specifically, we examined whether the average formant frequencies and EMG activity of the orbicularis oris superior (OOS) muscle differed significantly in the two lip tube conditions (FLT and RLT) under unaltered auditory feedback, relative to the reference task involving vowel production without a lip tube or auditory perturbation (Ref). These three conditions were compared, using linear mixed-effects models (R package lme4) with the following structure: [F2 ~ condition + 1|participant] and [RMSEMG ~ condition + 1|participant], where condition (3 levels: Ref; FLT with unaltered auditory feedback; and RLT with unaltered auditory feedback) was treated as a fixed effect and participant as a random effect. Pairwise contrasts were subsequently tested with Bonferroni corrections for multiple comparisons (R package multcomp).

Both lip tubes significantly affected vowel acoustics, leading to higher frequencies of the first two formants relative to the reference condition (average rise of 26 ± 1 Hz for F1, $p < .0001$, and 62 ± 3 Hz for F2, $p < .0001$, which corresponds to differences of 6% and 4% respectively) (see Figure S2A and S2B). From an articulatory and physiological perspective, vowel /ø/ was produced with deformable lip tubes with significantly increased EMG activity of the orbicularis oris muscle with respect to the reference condition (average increase of 76 ± 1.9 µV, $p < .0001$, which corresponds to to approximately a 130% increase) (see Figure S2C below). These consequences on speech acoustics and the level of articulatory effort can be regarded as a slight articulatory perturbation introduced by our experimental paradigm and as a limitation for our experiment – the diameter for the tubes, at rest, being probably slightly too large for the production of vowel /ø/ in some participants.

Nevertheless, the main goal of the present study—and of this experimental paradigm—was to manipulate the articulatory effort involved in lip-rounding movements and to compare speech adaptation to altered auditory feedback under two otherwise comparable speech production conditions, differing only in the amount of effort required to achieve the compensatory gesture. From this perspective, only very small acoustic differences were observed between the two lip-tube conditions (5 ± 1 Hz for F1, $p < .0001$, and 14 ± 3 Hz for F2, $p = .018$). Although statistically significant, these frequency differences can be considered negligible, representing about 1% variation for both F1 and F2, and falling below known perceptual difference limens (Mermelstein, 1978). At the articulatory level, however, the production of vowel /ø/ with the RLT already exhibited—as intended—significantly greater lip-muscle activity under unaltered auditory feedback, compared to the FLT condition.

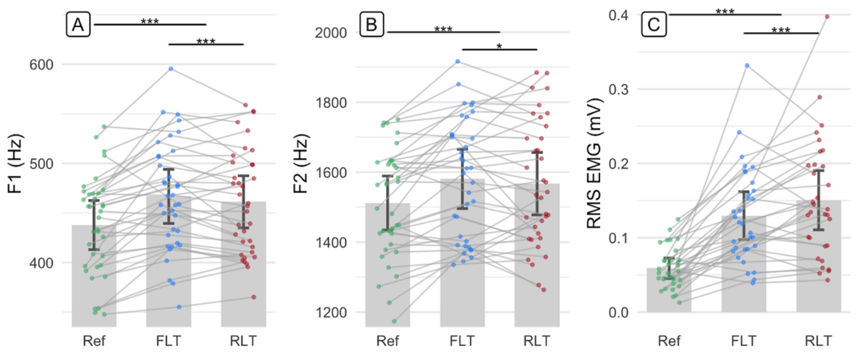

Supplementary Figure S2. Average frequency of the first two formants (F1, F2) and lip muscle activity (RMS_{EMG}) during the production of vowel /ø/ under unaltered auditory feedback, either naturally, without articulatory constraints (Ref), or with a very flexible (FLT) or more rigid (RLT) lip tube inserted between the lips. Error bars indicate 95% confidence intervals across participants (N=36 for acoustic measures; N=32 for EMG measures). Graphs A, B and C respectively show the average of F1, F2 and RMS_{EMG} values under unaltered auditory feedback for the 3 conditions.

Supplementary Material 3 - Absolute level of lip muscle activity

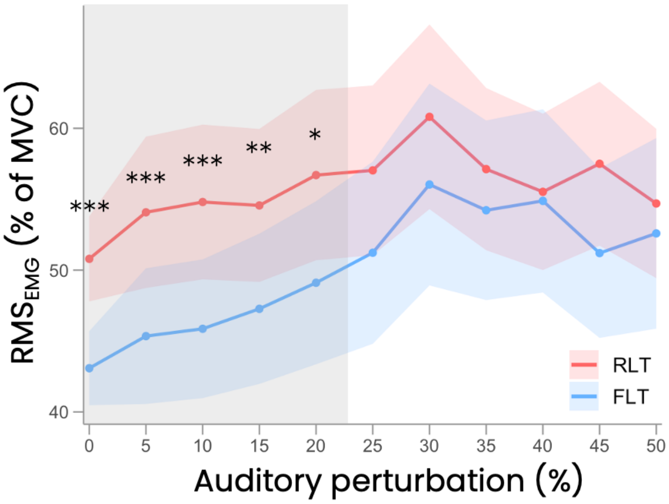

Supplementary Figure S3. Absolute level of lip muscle activity (RMS_{EMG}), during the production of vowel /ø/ under progressively increased auditory feedback perturbation (0% to 50% F2 upshift), with either a very flexible (FLT) or a more rigid (RLT) lip tube inserted between the lips. This EMG level is expressed as a percentage of the Maximum Voluntary Contraction (MVC) of the orbicularis oris muscle. Thick lines and shaded ribbons represent the mean and 95% confidence intervals across participants (N = 21) at each perturbation level. Grey areas highlight the perturbation levels at which significant differences were observed between the two lip tube conditions.

Supplementary Material 4 – Behavior of the 10 remaining participants

Additional insight can be gained from examining the compensatory behavior of the 10 participants who did not significantly recruit their lips to achieve acoustic compensation in both conditions, but in only one of them – either in FLT (N = 4) or in RLT (N = 6) (see Flowchart in Figure 3). These participants clearly used different strategies depending on the lip tube condition.

In all cases, a significant decrease in F2 was still observed for these participants, even in the conditions where they did not increase lip muscle activity. The F1 variations observed for the four participants who did not increase their lip muscle activity in the RLT condition (Figure 10, left panel), support the idea that in this condition, they rather relied on the backward movement of the tongue to compensate for the F2 upshift : indeed a joint decrease of F1 and F2 was observed in FLT, during the initial phase when lip muscle activity increases, whereas an increase of F1 rather accompanied the F2 decrease in RLT, where lip muscle activity rather decreased, as well as in the second phase of FLT where lip muscle activity also decreased after the initial increase. The same statement applies to the six participants who did not increase their lip muscle activity in the FLT condition (Figure 10, right panel).

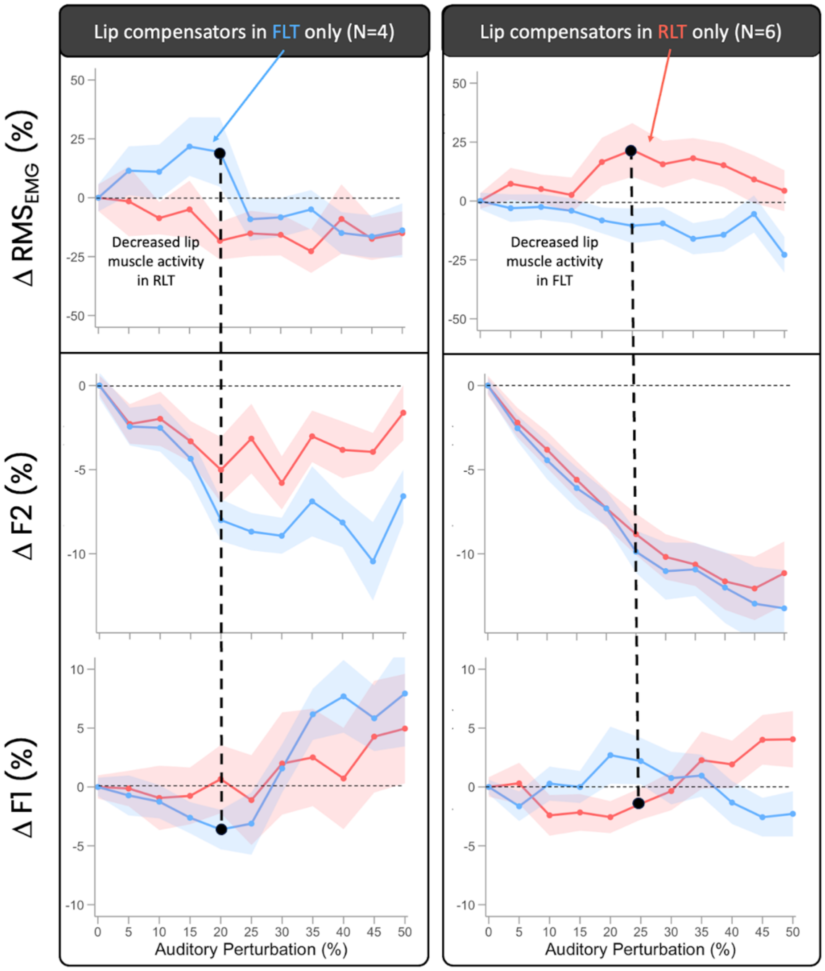

Supplementary Figure S4. Relative variation in lip muscle activity (ΔRMSEMG) and in the frequencies of the first two formants (ΔF1, ΔF2) during the production of vowel /ø/ under progressively increased auditory feedback perturbation (0% to 50% F2 upshift) relative to the values of these parameters under unaltered auditory feedback, with either a very flexible (FLT) or a more rigid (RLT) lip tube inserted between the lips, for the 10 participants who showed an increase in lip-muscle activity during compensation only in the FLT condition (N=4) or in the RLT condition (N=6). Thick lines and shaded ribbons represent the mean and 95% confidence intervals across participants at each perturbation level.

Supplementary Material 5 – Assessment of possible compensatory strategies in response to an F2 upward shift

In order to assess the articulatory strategies likely to be used to compensate for an F2 upward shift during the production of the French vowel /ø/, we used a simplified geometric representation of the vocal tract geometry of this vowel, inspired by the famous 4-tube model introduced by Fant (1960). This representation has been shown to properly account for the variations of the first three formants (F1, F2, F3) of the vowels (see in particular the Nomograms in Fant (1960)). The corresponding area function (representation of the cross-sectional area of the vocal tract from the glottis to the lips) of vowel /ø/ under normal condition is represented in Figure S5a (black line). The corresponding first three formants, computed with an acoustic harmonic model of the vocal tract (Badin & Fant, 1984) are: F1 = 410 Hz – F2 = 1475 Hz – F3 = 2332 Hz. These values are in the range of the formant values experimentally measured for vowel /ø/ in French speakers (Tubach, 1984) (F1= 381 ± 44 Hz – F2 = 1417 ± 106 Hz – F3 = 2235 ± 113 Hz). We then simulated the articulatory effect of the lip tube insertion (diameter 1.5 cm, cross-sectional area 1.77 cm^2) by increasing the lip area, as displayed in Figure S5a (red dotted line). As a consequence of this geometrical change the values of the first three formants become: F1 = 482 Hz – F2 = 1834 Hz – F3 = 2369 Hz. As observed in experimental data the introduction of the lip-tube induced an increase of F1 and F2, which makes the production closer to vowel /e/ (F1= 365 ± 31 Hz – F2 = 1961 ± 119 Hz – F3 = 2644 ± 107 Hz, for French speakers (Tubach, 1984)).

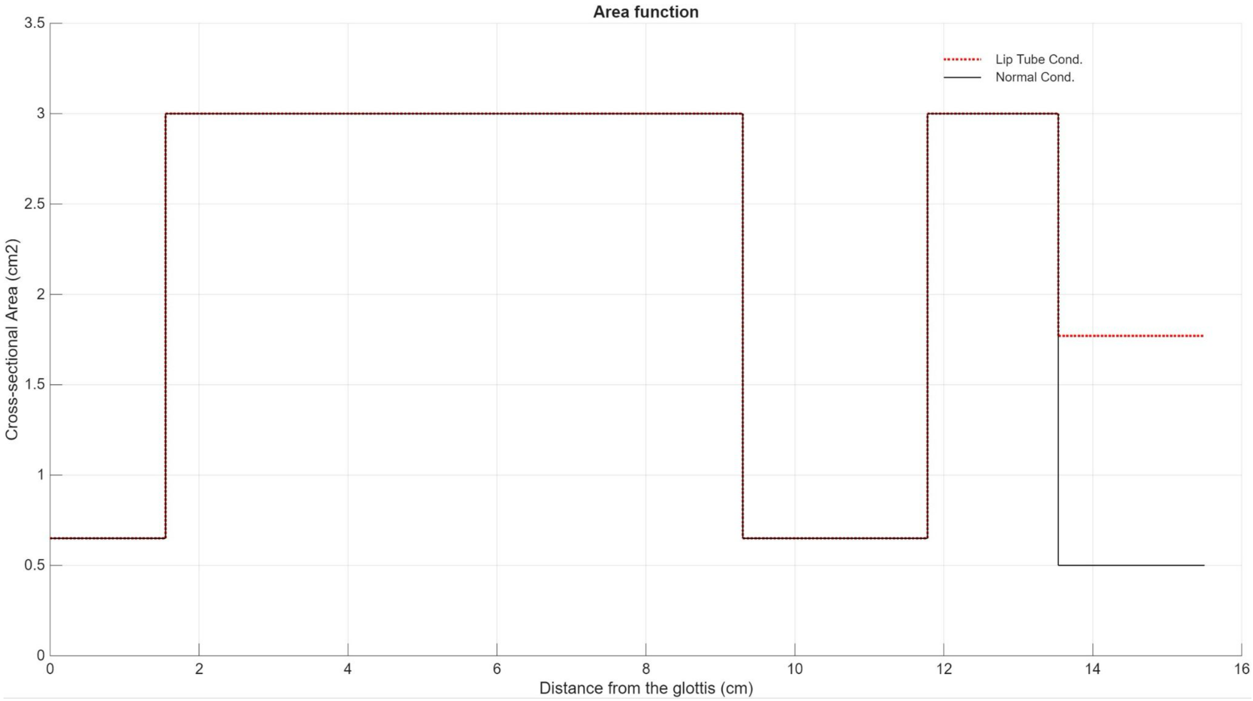

Supplementary Figure S5a. 4-tube models of the French vowel /ø/ under normal (black line) and lip tube (red dotted line) conditions.

We then assessed the effect on F1 and F2 of the three main geometrical changes induced by possible articulatory movements during the production of vowel /ø/ with the lip tube in place: change in the front/back position of the constriction, associated with tongue displacement; change in lip area; change in the laryngeal tube associated with a lowering/raising of the larynx. The results are depicted in Supplementary Figure S5b to S5e.

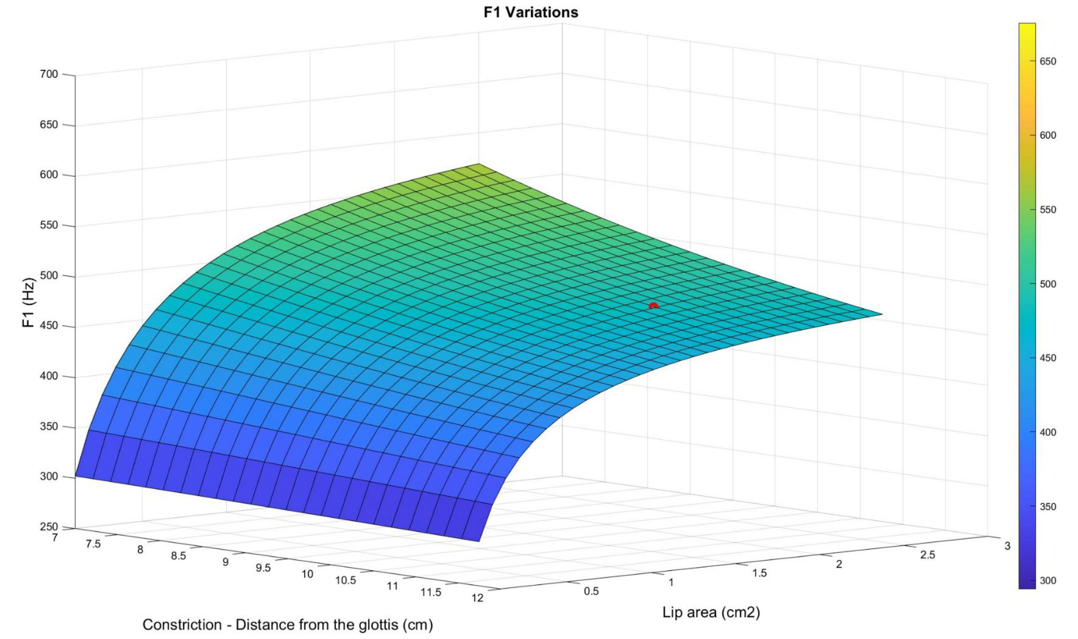

Supplementary Figure S5b. Effect on F1 of changes in constriction position and lip area for vowel /ø/ under the lip tube condition**.** The red circle shows the position of vowel /ø/ with the lip tube (lip area=1.77 cm^2; distance from the glottis for the constriction=10.5 cm; F1= 482 Hz)

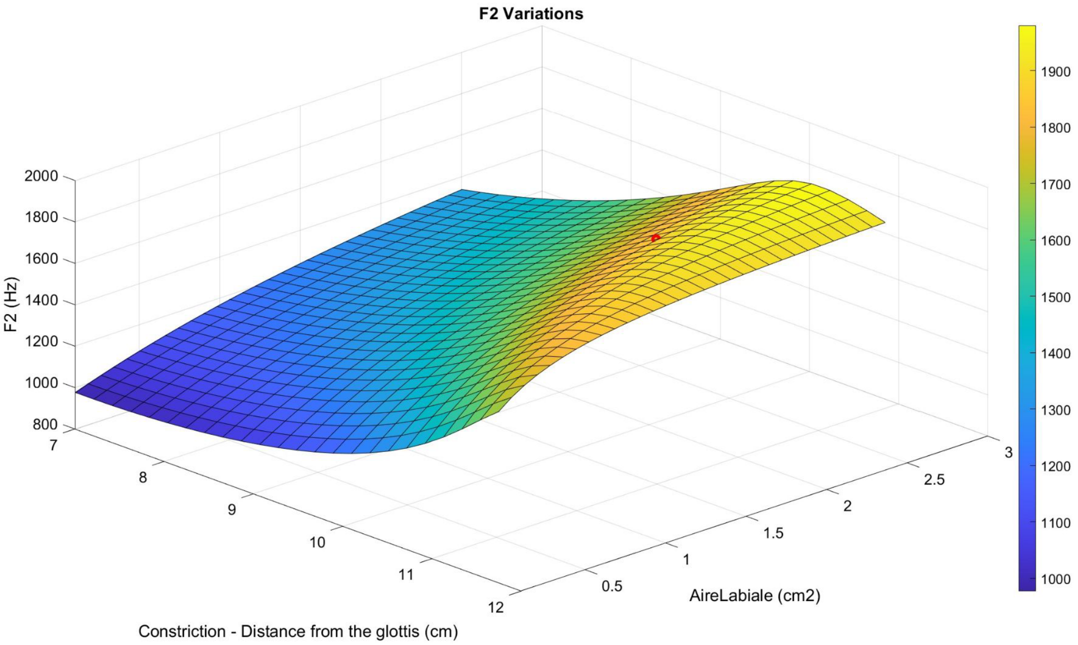

Supplementary Figure S5c. Effect on F2 of changes in constriction position and lip area for vowel /ø/ under the lip tube condition**.** The red circle shows the position of vowel /ø/ with the lip tube (lip area=1.77 cm^2; distance from the glottis for the constriction=10.5 cm; F2= 1834 Hz)

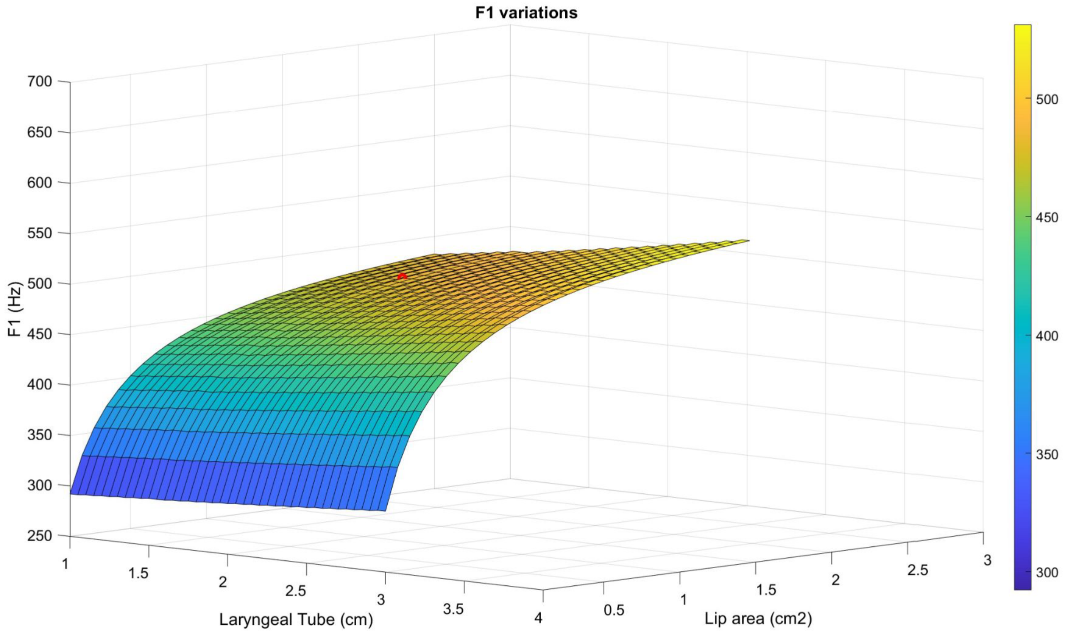

Supplementary Figure S5d. Effect on F1 of changes in larynx height and lip area for vowel /ø/ under the lip tube condition. Larynx lowering corresponds to an increase of the length of the laryngeal tube. (see Figure S6 for details)

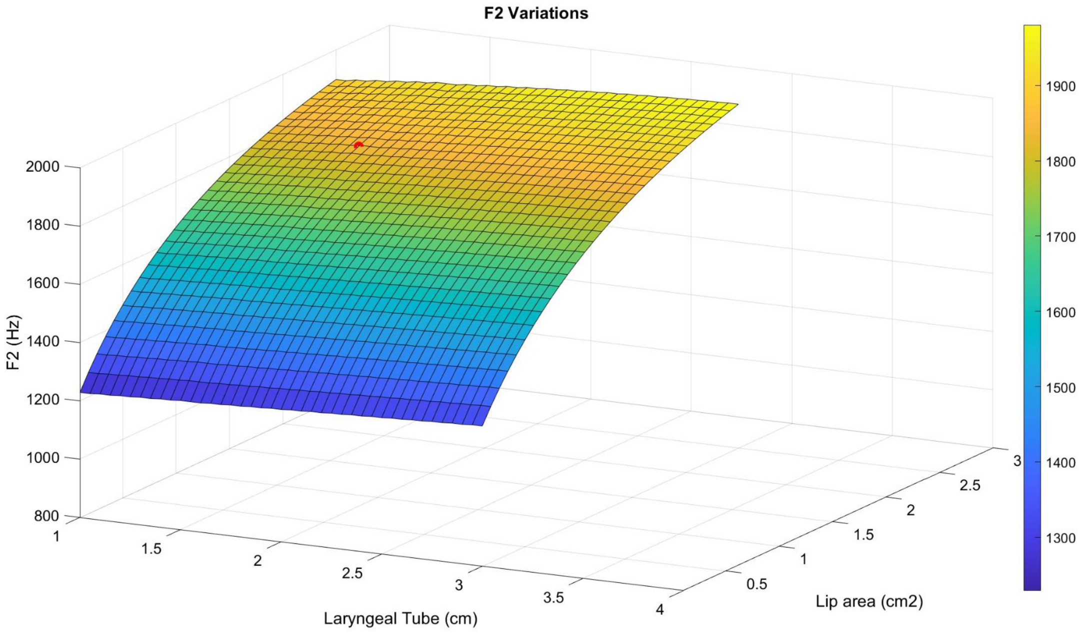

Supplementary Figure S5e. Effect on F2 of changes in larynx height and lip area for vowel /ø/ under the lip tube condition. Larynx lowering corresponds to an increase of the length of the laryngeal tube. (see Figure S7 for details)

Our results are in agreement with basic principles of the acoustic theory of vowel production (Fant, 1960). We observe that decreasing lip area induces a decrease of both F1 and F2. This effect is particularly strong for small lip areas. Moving the constriction backwards (i.e. moving the tongue backwards) also induces a decrease of F2 with a particularly strong effect in the range [11 cm 9 cm], but it induces an increase of F1. A lowering of the larynx also contributes to the decrease of F2. Importantly, it also induces a decrease of F1. The acoustic consequenceS of this movement are clearly less than the one of a decrease of the lip area or a backward movement of the tongue.

In sum, modeling vowel /ø/ with a 4-tube model is an efficient method to investigate the consequences of the major articulatory changes on the spectral characteristics of the vowel. It does not integrate other, more local, changes in the shape of the vocal tract, but these changes have been shown to essentially after formants F4 and F5, which are more related to speaker-specific characteristics and are not relevant for the perceptual characterization of the vowel (see for example Takemoto et al., 2010).

From this simulation work, we can conclude that, in response to an F2 upward shift, the compensation strategies that induce an increase of F1 involve a reduction of the lip area and a backward displacement of the constriction, with a larger weight for the constriction displacement. The backward tongue movement is the only possible articulatory strategy combining the required decrease of F2 with an increase of F1.

REFERENCES

Badin, P., & Fant, G. (1984). Notes on vocal tract computation, *STL QPSR 2(3)*, 53–108.

Tubach, J.P. (1989) La Parole et son traitement automatique [Speech automatic processing]; Masson, Paris.

Fant, G. (1960). *Acoustic theory of speech productions.* Mouton, The Hague.

Takemoto, H., Mokhtari, P., & Kitamura, T. (2010). Acoustic analysis of the vocal tract during vowel production by finite-difference time-domain method. *The Journal of the Acoustical Society of America*, *128*(6), 3724-3738.
